# Development and validation of combined Ki67 status prediction model for intrahepatic cholangiocarcinoma based on clinicoradiological features and MRI radiomics

**DOI:** 10.1007/s11547-023-01597-7

**Published:** 2023-02-11

**Authors:** Xianling Qian, Changwu Zhou, Fang Wang, Xin Lu, Yunfei Zhang, Lei Chen, Mengsu Zeng

**Affiliations:** 1 Department of Radiology, Zhongshan Hospital, Fudan University, No.180 Fenglin Rd, Shanghai, 200032 China; 2 Shanghai Institute of Medical Imaging, No.180 Fenglin Rd, Shanghai, 200032 China; 3 Department of Cancer Center, Zhongshan Hospital, Fudan University, No.180 Fenglin Rd, Shanghai, 200032 China; 4 Shanghai United Imaging Intelligence Co., Ltd, No.701 Yunjin Rd, Shanghai, 200232 China; 5 Central Research Institute, United Imaging Healthcare, No.2258 Chengbei Rd, Shanghai, 201807 China

**Keywords:** Intrahepatic cholangiocarcinoma, Ki67, Magnetic resonance imaging, Radiomics

## Abstract

**Purpose:**

Incidence and mortality of intrahepatic cholangiocarcinoma (ICC) have been increasing over the past few decades, and Ki67 is an adverse prognostic predictor and an attractive therapeutic target for ICC patients. Thus, we aim to develop and validate a combined Ki67 prediction model for ICC patients.

**Materials and methods:**

Preoperative contrast-enhanced MR images were collected from 178 patients with postoperative pathologically confirmed ICC, and randomly divided into training and validation cohorts in a ratio of 7:3 (124:54). A time-independent test cohort of 49 ICC patients was used for validation. Independent clinicoradiological features of Ki67 status were determined by multivariate analysis. Optimal radiomics features were selected by least absolute shrinkage and selection operator logistic regression and linear discriminant analysis was used to construct combined models. The prediction efficacy of combined model was assessed by receiver operating characteristics curve, and verified by its calibration, decision and clinical impact curves.

**Results:**

HBV (*p* = 0.022), arterial rim enhancement (*p* = 0.006) and enhancement pattern (*p* = 0.012) are independent clinicoradiological features. The radiomics model achieves good prediction efficacy in the training cohort (AUC = 0.860) and validation cohort (AUC = 0.843). The combined Ki67 prediction model incorporates clinicoradiological and radiomics features, and it yields desirable predictive efficiency in test cohort (AUC = 0.815). Decision curves and clinical impact curves further validate that the combined Ki67 prediction model can achieve net benefits in clinical work.

**Conclusion:**

The combined Ki67 model incorporating HBV, arterial rim enhancement, enhancement pattern and radiomics features is a potential biomarker in Ki67 prediction and stratification.

**Supplementary Information:**

The online version contains supplementary material available at 10.1007/s11547-023-01597-7.

## Introduction

Primary liver cancer (PLC) includes hepatocellular carcinoma (HCC), intrahepatic cholangiocarcinoma (ICC) and other rare type, and ICC accounts for 10–15% of PLC [1]. ICC originates from the intrahepatic secondary bile duct and has three gross pathological patterns: mass-forming (78%), periductal infiltrating (16%) and intraductal papillary (6%) subtypes, and the overall survival of patients with mass-forming ICC is shorter [2, 3]. The incidence and mortality of ICC have been increasing over the past few decades [4, 5]. Partial hepatectomy is an effective treatment [6], however, many patients with advanced ICC are inoperable due to delayed diagnosis [7]. Recently, molecular profiling has revealed subtypes of ICC [8], therefore, molecular targeted therapies are expected to improve the prognosis of ICC patients [9].

Ki67 protein is a nuclear antigen related to cell proliferation and positively correlated with cancer aggressiveness [10]. Some studies suggest that Ki67 is a poor prognostic predictor in patients with ICC [11, 12]. Besides, Ki67 is an attractive therapeutic target for malignant cancers [13]. For instance, Dinaciclib could suppress ICC growth by suppressing Ki67 protein [14]. Zhang et al*.* [15] also found that knockout of lncRNA CASC15 could suppress ICC progression by inhibiting Ki67 protein expression. Therefore, accurate prediction of Ki67 status in ICC patients is a predictor for treatment efficacy evaluation and outcome prediction. However, it is difficult to determine Ki67 status of ICC lesions by routine imaging and laboratory tests.

Currently, radiomics is defined as a high-throughput extraction of numerous image features from medical images, and independent features are applied to the construction of diagnostic, predictive and prognostic models [16]. Several studies have achieved good predictive efficiency in the Ki67 prediction of several malignant cancers, including HCC [17], breast cancer [18] and lung cancer [19]. However, the development and validation of Ki67 status prediction model for ICC lesions based on radiomics features has not yet been studied.

Serum carbohydrate antigen 19-9 (CA199) levels are often elevated in ICC patients, rather than elevated serum alpha-fetoprotein (AFP) levels like those in HCC patients. And typical mass-forming ICC lesions exhibit several imaging features like intrahepatic duct dilatation, hepatic capsular retraction and gradual and filling enhancement pattern. Therefore, preoperative diagnosis of ICC is not difficult, whereas, to date, there is no model to predict the Ki67 status of ICC lesions preoperatively. In our study, we aimed to develop and validate a combined Ki67 prediction model for mass-forming ICCs incorporating clinicoradiological features and MRI radiomics. Importantly, the combined Ki67 prediction model will be further validated by a time-independent test cohort.

## Materials and methods

### Patients

**Fig. 1:**
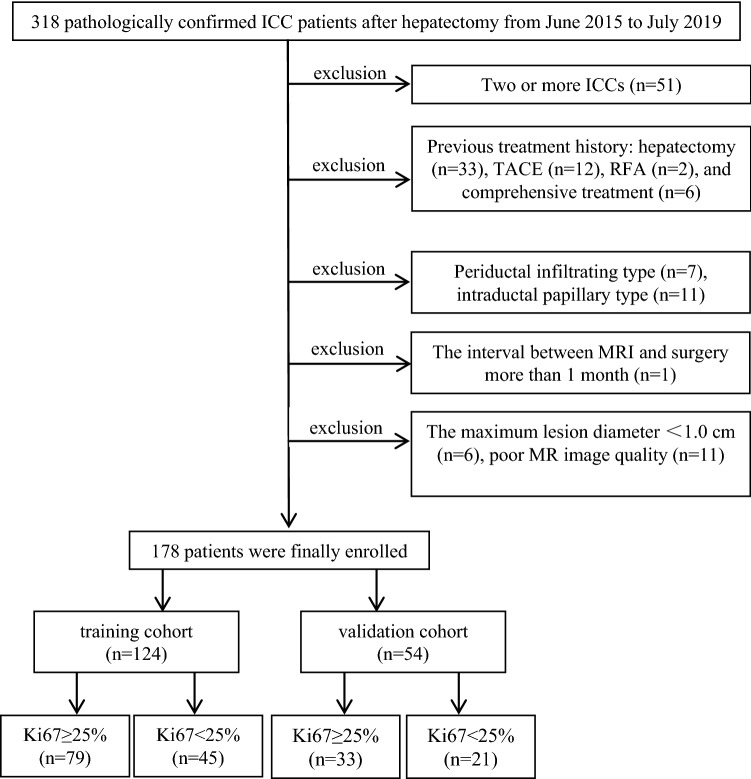
Study flowchart of the enrolled patients

This retrospective-prospective study was approved by the Zhongshan Hospital, Fudan University (Ethics approval No. B2021-325R) ethics committees, and patient informed consent was waived. From June 2015 to July 2019, 178 patients with postoperative pathologically confirmed ICC from the Zhongshan Hospital were randomly divided into the training (*n* = 124, 79 high Ki67 status group and 45 low Ki67 status group) and validation cohorts (*n* = 54, 33 high Ki67 status group and 21 low Ki67 status group) in a ratio of 7:3. From August 2019 to April 2022, a test cohort of postoperative pathologically confirmed ICC patients (*n* = 49, 28 high Ki67 status group and 21 low Ki67 status group) from the Zhongshan Hospital was prospective grouped with the same inclusion criteria, and baseline clinicoradiological features of ICC patients in test cohort are shown in Table S1. The main inclusion criteria (Fig. 1): (a) single lesion with longest diameter ≥ 1.0 cm; (b) without previous treatment history of PLC; (c) complete histopathological description of ICC lesion; (d) all patients underwent MRI examination within 1 month before hepatectomy; (e) adequate MR image quality.

### Clinical features retrieval

The demographic data, preoperative serum AFP, carcinoembryonic antigen (CEA), CA199, history of hepatitis B virus (HBV) serum markers and HBV-DNA loads were retrospectively retrieved (Table 1). All PLC samples were sampled using 7-point baseline sampling protocol [20]. Histopathological features including lesion number, Edmondson-Steiner grade and Ki67 status were evaluated by two experienced abdominal pathologists. Anti-human Ki67 rabbit monoclonal antibodies (Maixin Biotech Co., Ltd, Fuzhou, China) were used with a dilution of 1:50 in immunohistochemistry, and the Ki67 labeling index (LI) was recorded. We classified ICC lesions into low Ki67 status group (Ki67 LI < 25%) and high Ki67 status group (Ki67 LI ≥ 25%) as previous studies [11, 21].

**Table 1:** Baseline clinicoradiological features of ICC patients in training and validation cohorts

Features	Training cohort (*n* = 124)			Validation cohort (*n* = 54)			*p*-Inter
	Ki67 < 25% (*n* = 45)	Ki67 ≥ 25% (*n* = 79)	*p*-Intra	Ki67 < 25% (*n* = 21)	Ki67 ≥ 25% (*n* = 33)	*p*-Intra	
*Clinical features*							
Age (years)^a^	62.244 (9.759)	59.443 (11.690)	0.176	63.238 (10.616)	59.212 (11.567)	0.204	0.861
*Gender*			0.089			0.076	0.362
Male	29 (64.4)	62 (78.5)		11 (52.4)	25 (75.8)		
Female	16 (35.6)	17 (21.5)		10 (47.6)	8 (24.2)		
*HBV*			0.008			0.752	0.299
Negative	30 (66.7)	33 (41.8)		13 (61.9)	19 (57.6)		
Positive	15 (33.3)	46 (58.2)		8 (38.1)	14 (42.4)		
*AFP*			0.458			1.000	0.076
< 20 ng/ml	42 (93.3)	69 (87.3)		17 (81.0)	26 (78.8)		
≥ 20 ng/ml	3 (6.7)	10 (12.7)		4 (19.0)	7 (21.2)		
*CEA*			0.092			1.000	0.798
< 5 ng/ml	34 (75.6)	69 (87.3)		17 (81.0)	27 (81.8)		
≥ 5 ng/ml	11 (24.4)	10 (12.7)		4 (19.0)	6 (18.2)		
CA199			0.745			0.851	0.608
< 34 U/ml	26 (57.8)	48 (60.8)		12 (57.1)	18 (54.5)		
≥ 34 U/ml	19 (42.2)	31 (39.2)		9 (42.9)	15 (45.5)		
*Edmondson-Steiner grade*			0.006			0.177	0.134
I–II	25 (55.6)	24 (30.4)		8 (38.1)	7 (21.2)		
II–IV	20 (44.4)	55 (69.6)		13 (61.9)	26 (78.8)		
*MR imaging features*							
Tumor size(mm)^b^	34.9 (27.35–46.70)	44.5 (24.50–59.40)	0.307	49.5 (30.45–67.25)	45.8 (30.85–59.60)	0.613	0.183
*Tumor morphology*			0.611			0.065	0.525
(Hemi-)spherical and oval	14 (31.1)	31 (39.2)		10 (47.6)	14 (42.4)		
Lobulated	22 (48.9)	32 (40.5)		4 (19.0)	15 (45.5)		
Irregular	9 (20.0)	16 (20.3)		7 (33.3)	4 (12.1)		
*SI on T1WI*			0.159			–	0.231
Low	45 (100.0)	75 (94.9)		21 (100.0)	33 (100.0)		
Moderate	0 (0.0)	3 (3.8)		0 (0.0)	0 (0.0)		
High	0 (0.0)	1 (1.3)		0 (0.0)	0 (0.0)		
*SI on T2WI-FS*			0.560			1.000	0.602
Low	0 (0.0)	1 (1.3)		0 (0.0)	0 (0.0)		
Moderate	1 (2.2)	3 (3.8)		0 (0.0)	1 (3.0)		
High	44 (97.8)	75 (94.9)		21 (100.0)	32 (97.0)		
*Target sign on T2WI-FS*			0.243			0.686	0.286
Negative	24 (54.5)	49 (65.3)		14 (66.7)	23 (71.9)		
Positive	20 (45.5)	26 (34.7)		7 (33.3)	9 (28.1)		
*Target sign on DWI*			0.465			0.801	0.457
Negative	22 (48.9)	44 (55.7)		12 (57.1)	20 (60.6)		
Positive	23 (51.1)	35 (44.3)		9 (42.9)	13 (39.4)		
*Intrahepatic duct dilatation*			0.877			0.594	0.315
Negative	29 (64.4)	52 (65.8)		13 (61.9)	18 (54.5)		
Positive	16 (35.6)	27 (34.2)		8 (38.1)	15 (45.5)		
*Hepatic capsular retraction*			0.904			0.554	0.141
Negative	25 (55.6)	43 (54.4)		13 (61.9)	23 (69.7)		
Positive	20 (44.4)	36 (45.6)		8 (38.1)	10 (30.3)		
*Visible vessel penetration*			0.765			0.480	0.755
Negative	17 (37.8)	32 (40.5)		9 (42.9)	11 (33.3)		
Positive	28 (62.2)	47 (59.5)		12 (57.1)	22 (66.7)		
*Peripheral hepatic enhancement*			0.933			0.898	0.162
Negative	22 (48.9)	38 (48.1)		8 (38.1)	12 (36.4)		
Positive	23 (51.1)	41 (51.9)		13 (61.9)	21 (63.6)		
*Arterial rim enhancement on AP*			0.057			0.878	0.834
Negative	14 (31.1)	13 (16.5)		5 (23.8)	6 (18.2)		
Positive	31 (68.9)	66 (83.5)		16 (76.2)	27 (81.8)		
*Complete rim on AP*			0.322			0.280	0.597
Negative	16 (51.6)	27 (40.9)		8 (50.0)	9 (33.3)		
Positive	15 (48.4)	39 (59.1)		8 (50.0)	18 (66.7)		
*Enhancement pattern*			0.143			0.568	0.856
Gradual and filling	37 (82.2)	57 (72.2)		15 (71.4)	27 (81.8)		
Arterial and persistent	6 (13.3)	9 (11.4)		3 (14.3)	2 (6.1)		
Wash-in and wash-out	2 (4.4)	13 (16.5)		3 (14.3)	4 (12.1)		
*LI-RADS*			0.452			0.381	0.716
LR-3	1 (2.2)	0 (0.0)		0 (0.0)	0 (0.0)		
LR-4	1 (2.2)	3 (3.8)		0 (0.0)	1 (3.0)		
LR-5	2 (4.4)	6 (7.6)		3 (14.3)	2 (6.1)		
LR-M	41 (91.1)	69 (87.3)		18 (85.7)	30 (90.9)		
LR-TIV	0 (0.0)	1 (1.3)		0 (0.0)	0 (0.0)		

Unless otherwise stated, data are shown as number of patients with percentage in parentheses

^a^Data are means with standard deviation in parentheses

^b^Data are medians with interquartile ranges in parentheses

### Gd-DTPA MR acquisition protocol

Contrast-enhanced MRI was performed by intravenous injection of 0.1 mmol/kg Gd-DTPA, followed immediately by a 20 ml saline flush at 2 ml/s. Taking 3.0 T uMR 770 scanner (United Imaging Healthcare, Shanghai, China) as an example, MR sequences involved in this study include axial T2-weighted imaging with fat suppression (T2WI-FS), diffusion-weighted imaging (DWI, with *b* values of 0, 50, 500 s/mm^2^), axial pre-contrast quick three-dimensional T1-weighted imaging (quick3d T1WI) with fat suppression and post-contrast dynamic-enhanced quick 3d T1WI at arterial phase (AP, 20–30 s), portal venous phase (PVP, 60–70 s) and delayed phase (DP, 180 s) (Table S2).

### Imaging features analysis

Imaging features were assessed independently by 2 blinded and experienced abdominal radiologists (C.W.Z. and X.L. with 10 and 15 years of experience, respectively). In case of any discrepancy, a consensus was generated after discussion. Imaging features including: (a) tumor size; (b) tumor morphology; (c) signal intensity (SI) on T1WI, T2WI-FS and DWI images; (d) target sign (peripheral hyperintense with central isointense/hypointense) [22]; (e) rim enhancement on AP (complete and incomplete rim); (f) enhancement pattern; (g) the liver imaging reporting and data system (LI-RADS) (Version 2018) [23]; (h) intrahepatic duct dilatation; (i) hepatic capsular retraction (retraction of hepatic capsular adjacent to the lesion); (j) visible vessel penetration [24]; (k) peripherally hepatic enhancement (peritumoral enhancement on any phase).

### Radiomics analysis

#### Tumor segmentation

The tumor segmentation was performed in the ITK-SNAP software. Volumes of interests (VOIs) were manually delineated based on 6 MR sequences (DWI with *b* value of 500 s/mm^2^, T2WI-FS, pre-T1WI, AP, PVP and DP, respectively) by an abdominal radiologist with 5 years of experience (X.L.Q.) and checked by a senior abdominal radiologist (X.L.). Besides, 30 MR images of ICC lesions were randomly selected and delineated again by X.L.Q. to assess the test–retest reliability. Blinded to segmentations delineated by X.L.Q., these 30 MR images of ICC lesions were delineated again by C.W.Z. to assess the inter-observer variability.

#### Feature extraction

To reduce heterogeneity among MR images, all images were resampled to an isotropic voxel size (1 × 1 × 1 mm^3^) using bilinear interpolation, and intensities were normalized with a fixed bin width. Images were then normalized by z-score to obtain a standard normal distribution of image intensity. The extraction of radiomics features was performed by the uAI Research Portal (Version: 20210730), in which PyRadiomics (https://pyradiomics.readthedocs.io/en/v3.0.1/) was embedded. 2600 radiomics features were extracted from each sequence, and these features were classified into first-order statistics, shape-based features, texture features, and high-order features.

#### Feature selection

Firstly, extracted radiomics features were applied with a *z*-score $\left( {y_{i} = \frac{{x_{i} - \overline{x}}}{\sigma \left( x \right)}} \right)$ normalization to eliminate index dimension difference. Secondly, features with intraclass correlation coefficients ≥ 0.75 in both test–retest and inter-observer settings were considered as reproducible radiomics features and were chosen for further analysis. Finally, the correlation analysis, multicollinearity analysis and least absolute shrinkage and selection operator (LASSO) methods were performed to select optimal prediction features (Table S3, Figure S1).

#### Model construction

Clinical model and imaging model were constructed by corresponding independent clinicoradiological predictors. For models based on single or multiple MR sequences and fusion models, linear discriminant analysis (LDA) was used to find the best linear combination of the above optimal prediction features to maximize the discrimination between patients with high and low Ki67 status. On the basis that LDA has a certain classification ability, taking the LDA results as the input feature of logistic regression (LR) and random forest (RF) to further accurately identify patients with high or low Ki67 status, and the classification threshold is 0.5.

#### Model evaluation and verification

**Fig. 2:**
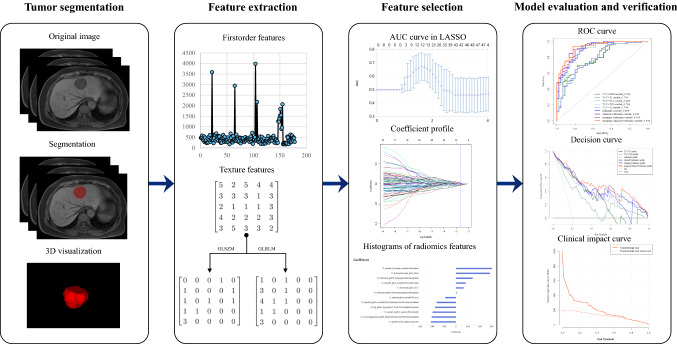
Study flowcharts of radiomics analysis

Receiver operating characteristics curves (ROC) were plotted and the area under curve (AUC), sensitivity, specificity, accuracy, precision and F1-score were calculated to evaluate the performance of models. Delong test was used to compare the predictive efficiency between models, and we applied the Benjamini–Hochberg method to correct the false discovery rate (FDR) [25]. Hosmer–Lemeshow test was performed to evaluate the consistency between actual Ki67 status and predicted Ki67 status, and calibration curve was plotted. Decision curve and clinical impact curve were plotted to verify the clinical practicability of models by quantifying the net benefits at different risk thresholds. The confusion matrixes of the fusion models in three models were plotted. The workflow of the above radiomics analysis is shown in Fig. 2.

#### Correlation analysis

Radiomics features extracted from pre-T1WI, PVP and DP sequences were correlated with HBV, arterial rim enhancement on AP, enhancement pattern and Ki67, respectively, and heatmaps were performed. Because the different ranges of features, correlation coefficients were calculated for continuous versus binary variables, continuous variables, and non-continuous variables by using Biserial, Pearson, and Spearman correlation analyses, respectively.

### Statistical analysis

Student’s *t* test was used when variables were normal distribution, and Mann–Whitney *U* test was used when variables were non-normal distribution for continuous variables and chi-square test was used for qualitative variables to analyze whether there were statistically significant differences. Univariate and multivariate analysis were used for the selection of independent predictor. Statistical analysis was performed with R software (version 4.1.1). All statistical tests were two-sided, and *p* value lower than 0.05 were considered statistically significant.

## Results

### Performance of clinicoradiological features

**Fig. 3:**
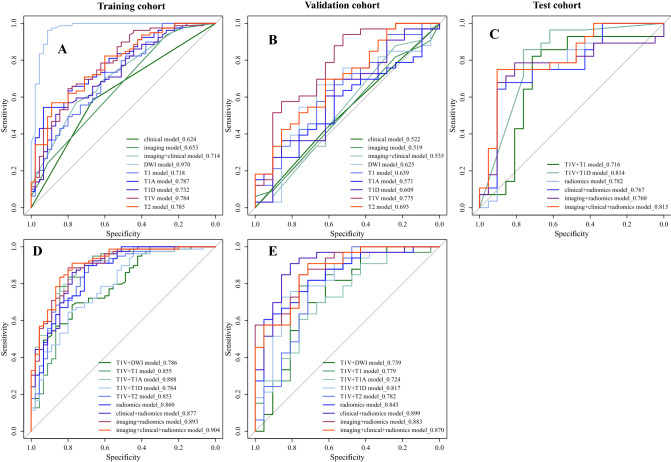
Comparison of receiver operating characteristics (ROC) curves for Ki67 status prediction in training (**A**, **D**), validation (**B**, **E**) and test (**C**) cohorts by logistic regression

124 and 54 patients were divided into training and validation cohorts, and baseline clinicoradiological features are shown in Table 1. At the univariate analysis, gender, HBV, CEA, arterial rim enhancement on AP and enhancement pattern are significantly related to Ki67 status. The multivariate analysis shows HBV, arterial rim enhancement on AP and enhancement pattern are independent predictors of Ki67 status in ICC patients (Table 2). In the training cohort and validation cohort, the clinical model constructed with HBV and imaging model constructed with arterial rim enhancement on AP and enhancement pattern both exhibit poor predictive efficiency. The clinical + imaging model also shows an unsatisfactory predictive efficiency (AUC_training_ = 0.714, AUC_validation_ = 0.535) (Table 3, Fig. 3A, B). Example of representative clinicoradiological features of ICC with high Ki67 status are shown in Figure S2.

**Table 2:** Univariate and multivariate analyses of clinicoradiological features related with Ki67 status in ICC

Features	Univariate			Multivariate		
	*b*-value	*p*-value	OR (95% CI)	*b*-value	*p*-value	OR (95% CI)
Age	− 0.024	0.177	0.977 (0.943–1.010)			
Gender	− 0.699	0.092	0.497 (0.219–1.123)	− 0.335	0.493	0.716 (0.275–1.890)
**HBV**	**1.025**	**0.009**	**2.788 (1.315–6.104)**	**1.019**	**0.022**	**2.770 (1.176–6.798)**
AFP	0.708	0.303	2.029 (0.582–9.430)			
CEA	− 0.803	0.097	0.448 (0.170–1.162)	− 0.781	0.167	0.458 (0.149–1.398)
CA199	− 0.124	0.745	0.884 (0.420–1.871)			
Edmondson− Steiner grade	1.052	0.007	2.865 (1.351–6.194)			
Tumor size	0.006	0.398	1.006 (0.992–1.022)			
Tumor morphology	− 0.146	0.566	0.864 (0.523–1.425)			
SI on T1WI	15.325	0.987	4,524,206.867 (0-NA)			
SI on T2WI-FS	− 0.848	0.403	0.428 (0.024–2.171)			
Target sign on T2WI-FS	− 0.451	0.245	0.637 (0.296–1.364)			
Target sign on DWI	− 0.273	0.466	0.761 (0.364–1.586)			
Intrahepatic duct dilatation	− 0.061	0.877	0.941 (0.439–2.049)			
Hepatic capsular retraction	0.045	0.904	1.047 (0.502–2.197)			
Visible vessel penetration	− 0.115	0.765	0.892 (0.416–1.883)			
Peripheral hepatic enhancement	0.032	0.933	1.032 (0.495–2.151)			
**Arterial rim enhancement on AP**	**0.830**	**0.061**	**2.293 (0.962–5.521)**	**1.946**	**0.006**	**6.998 (1.935–33.724)**
Complete rim on AP	0.432	0.324	1.541 (0.653–3.667)			
**Enhancement pattern**	**0.526**	**0.093**	**1.691 (0.951–3.315)**	**1.140**	**0.012**	**3.127 (1.394–8.558)**
LI-RADS	0.064	0.862	1.066 (0.490–2.183)			

Bold values are features with *p* < 0.05 in univariate and multivariate analyses

**Table 3:** The performance of clinicoradiological features, radiomics features and the combined predictive models for predicting Ki67 status in ICC patients

Models	Classifiers	Features	AUC (95% CI)	Sensitivity	Specificity	Accuracy	Precision	F1-score
Clinical model	LR (TD/VD)	1	0.624 (0.536–0.713) 0.522 (0.385–0.658)	1.000/1.000	0.000/0.000	0.363/0.611	0.363/0.611	0.533/0.759
	RF (TD/VD)		0.624 (0.536–0.713) 0.522 (0.385–0.658)	1.000/1.000	0.000/0.000	0.363/0.611	0.363/0.611	0.533/0.759
Imaging model	LR (TD/VD)	2	0.653 (0.571–0.735)/0.519 (0.389–0.650)	0.975/0.909	0.178/0.048	0.685/0.574	0.675/0.600	0.798/0.723
	RF (TD/VD)		0.653 (0.571–0.735) 0.456 (0.326–0.586)	0.937/0.879	0.267/0.095	0.694/0.574	0.692/0.604	0.796/0.716
Clinical + imaging model	LR (TD/VD)	3	0.714 (0.626–0.803) 0.535 (0.376–0.694)	0.975/0.909	0.200/0.095	0.694/0.593	0.681/0.612	0.802/0.732
	RF (TD/VD)		0.717 (0.629–0.805) 0.530 (0.416–0.625)	0.975/0.909	0.200/0.095	0.694/0.593	0.681/0.612	0.802/0.732
DWI model	LR (TD/VD)	12	0.970 (0.938–1.000)/0.625 (0.464–0.786)	0.949/0.697	0.911/0.524	0.935/0.630	0.949/0.697	0.949/0.697
	RF (TD/VD)		0.979 (0.958–1.000) 0.615 (0.456–0.775)	0.975/0.636	0.889/0.667	0.944/0.648	0.939/0.750	0.957/0.689
T1 model	LR (TD/VD)	11	0.718 (0.623–0.812) 0.639 (0.485–0.794)	0.899/0.848	0.333/0.286	0.694/0.630	0.703/0.651	0.789/0.737
	RF (TD/VD)		0.756 (0.666–0.846) 0.650 (0.495–0.805)	1.000/0.939	0.289/0.238	0.742/0.667	0.712/0.660	0.832/0.775
T1A model	LR (TD/VD)	13	0.787 (0.707–0.868) 0.571 (0.416–0.727)	0.873/0.758	0.422/0.286	0.710/0.574	0.726/0.625	0.793/0.685
	RF (TD/VD)		0.846 (0.780–0.913) 0.608 (0.450–0.766)	0.911/0.788	0.422/0.238	0.734/0.574	0.735/0.619	0.814/0.693
T1V model	LR (TD/VD)	12	0.784 (0.700–0.869) 0.775 (0.640–0.909)	0.949/0.848	0.444/0.571	0.766/0.741	0.750/0.757	0.838/0.800
	RF (TD/VD)		0.816 (0.738–0.893) 0.773 (0.640–0.906)	0.949/0.939	0.444/0.524	0.766/0.778	0.750/0.756	0.838/0.838
T1D model	LR (TD/VD)	11	0.732 (0.640–0.823) 0.609 (0.449–0.769)	0.962/0.879	0.267/0.286	0.710/0.648	0.697/0.659	0.808/0.753
	RF (TD/VD)		0.769 (0.687–0.852) 0.589 (0.428–0.749)	0.962/0.818	0.267/0.286	0.710/0.611	0.697/0.643	0.808/0.720
T2 model	LR (TD/VD)	17	0.785 (0.703–0.867) 0.693 (0.547–0.838)	0.873/0.879	0.444/0.333	0.718/0.667	0.734/0.674	0.798/0.763
	RF (TD/VD)		0.821 (0.749–0.893) 0.686 (0.540–0.832)	0.823/0.848	0.578/0.381	0.734/0.667	0.774/0.683	0.798/0.757
T1V + DWI model	LR (TD/VD)	24	0.786 (0.703–0.868) 0.739 (0.590–0.888)	0.899/0.818	0.422/0.571	0.726/0.722	0.732/0.750	0.807/0.783
	RF (TD/VD)		0.845 (0.776–0.915) 0.735 (0.592–0.879)	0.949/0.818	0.422/0.524	0.758/0.704	0.743/0.730	0.833/0.771
T1V + T1 model	LR (TD/VD)	23	**0.855 (0.776–0.934)** **0.779 (0.640–0.918)**	0.949/0.848	0.667/0.571	0.847/0.741	0.833/0.757	0.888/0.800
	RF (TD/VD)		**0.897 (0.840–0.955)** **0.815 (0.699–0.930)**	0.949/0.879	0.667/0.571	0.847/0.759	0.833/0.763	0.888/0.817
T1V + T1A model	LR (TD/VD)	22	0.888 (0.826–0.950) 0.724 (0.583–0.866)	0.911/0.788	0.667/0.476	0.823/0.667	0.828/0.703	0.868/0.743
	RF (TD/VD)		0.904 (0.851–0.957) 0.716 (0.574–0.857)	0.911/0.788	0.711/0.476	0.839/0.667	0.847/0.703	0.878/0.743
T1V + T1D model	LR (TD/VD)	19	**0.784 (0.700–0.869)** **0.817 (0.694–0.939)**	0.949/0.909	0.444/0.571	0.766/0.778	0.750/0.769	0.838/0.833
	RF (TD/VD)		**0.814 (0.735–0.892)** **0.808 (0.684–0.932)**	0.949/0.727	0.444/0.857	0.766/0.778	0.750/0.889	0.838/0.800
T1V + T2 model	LR (TD/VD)	25	0.853 (0.781–0.926) 0.782 (0.642–0.922)	0.911/0.939	0.622/0.476	0.806/0.759	0.809/0.738	0.857/0.827
	RF (TD/VD)		0.874 (0.812–0.937) 0.766 (0.618–0.914)	0.899/0.939	0.689/0.476	0.823/0.759	0.835/0.738	0.866/0.827
Radiomics model	LR (TD/VD)	26	**0.860 (0.799–0.934)** **0.843 (0.756–0.933)**	0.899/0.939	0.644/0.667	0.806/0.833	0.816/0.816	0.855/0.873
	RF (TD/VD)		**0.905 (0.850–0.960)** **0.894 (0.806–0.982)**	0.937/0.939	0.622/0.667	0.823/0.833	0.813/0.816	0.871/0.873
Clinical + radiomics model	LR (TD/VD)	27	**0.877 (0.813–0.942)** **0.899 (0.809–0.989)**	0.911/0.909	0.689/0.810	0.831/0.870	0.837/0.882	0.873/0.896
	RF (TD/VD)		**0.892 (0.833–0.951)** **0.895 (0.801–0.989)**	0.911/0.909	0.689/0.810	0.831/0.870	0.837/0.882	0.873/0.896
Imaging + radiomics model	LR (TD/VD)	28	**0.893 (0.834–0.951)** **0.883 (0.795–0.971)**	0.911/0.879	0.622/0.667	0.806/0.796	0.809/0.806	0.857/0.841
	RF (TD/VD)		**0.911 (0.859–0.962)** **0.870 (0.775–0.965)**	0.886/0.788	0.778/0.762	0.847/0.778	0.875/0.839	0.881/0.812
Clinical + imaging + radiomics model	LR (TD/VD)	29	**0.904 (0.849–0.960)** **0.870 (0.775–0.965)**	0.924/0.879	0.689/0.714	0.839/0.815	0.839/0.829	0.880/0.853
	RF (TD/VD)		**0.920 (0.871–0.969)** **0.862 (0.763–0.961)**	0.911/0.818	0.778/0.762	0.863/0.796	0.878/0.844	0.894/0.831
Clinical + imaging model	LR (test)	3	0.651 (0.502–0.782)	0.857	0.333	0.633	0.632	0.727
	RF (test)		0.638 (0.488–0.770)	0.893	0.238	0.612	0.610	0.725
T1V + T1 model	LR (test)	23	0.716 (0.550–0.882)	0.857	0.619	0.755	0.750	0.800
	RF (test)		0.816 (0.693–0.940)	0.857	0.619	0.755	0.750	0.800
T1V + T1D model	LR (test)	19	0.814 (0.689–0.939)	0.964	0.619	0.816	0.771	0.857
	RF (test)		0.792 (0.680–0.904)	0.964	0.619	0.816	0.771	0.857
Radiomics model	LR (test)	26	0.782 (0.639–0.925)	0.750	0.619	0.694	0.724	0.737
	RF (test)		0.813 (0.693–0.932)	0.750	0.571	0.673	0.700	0.724
Clinical + radiomics model	LR (test)	27	0.767 (0.625–0.909)	0.750	0.667	0.714	0.750	0.750
	RF (test)		0.806 (0.681–0.931)	0.750	0.667	0.714	0.750	0.750
Imaging + radiomics model	LR (test)	28	0.760 (0.612–0.909)	0.786	0.714	0.755	0.786	0.786
	RF (test)		0.771 (0.628–0.914)	0.750	0.810	0.776	0.840	0.792
Clinical + imaging + radiomics model	LR (test)	29	**0.815 (0.688–0.941)**	**0.750**	**0.767**	**0.714**	**0.750**	**0.750**
	RF (test)		0.807 (0.680–0.934)	0.714	0.810	0.755	0.833	0.769

Bold values are models with stable and/or desirable predictive performance

### Performance of radiomics features using single MR sequence

Totally, 76 robust radiomics features were selected from 6 single MR sequences (Figure S1, Table S3 and S4), and the AUCs of 6 single MR sequence models constructed with robust radiomics features are displayed (Table 3, Fig. 3A, B). Although all single MR sequence models show poor specificity, which prompts single MR sequence model predictive efficiency is unreliable, T1, T1V and T1D models yield stable AUCs between training and validation cohorts, and T1V model shows the most stable predictive efficiency with Δ_AUC_ = 0.009. Therefore, multiple-sequence models were constructed based on above three single-sequence models.

### Performance of radiomics features using multiple MR sequence

Two-sequence models have good predictive efficiency in training and validation cohorts. Among them, T1V + T1 and T1V + T1D models show stable predictive efficacy and higher AUCs in validation than training cohort. Thereby, the final three-sequence radiomics model incorporates T1, T1V and T1D models, and it shows a satisfying predictive performance (AUC_training_ = 0.860, AUC_validation_ = 0.843) (Table 3, Fig. 3D, E). And the final radiomics model performs better than T1V + T1D (FDR *p* = 0.018) model in the training cohort (Table 4). However, all of these three models show unsatisfying predictive performance in test cohort (AUC = 0.716–0.814) (Table 3 and Fig. 3C).

**Table 4:** The comparison of models in training, validation, and test cohorts

Models	Classifiers	*p*-train	*p*-validation	*p*-test
Radiomics model versus T1V + T1 model	LR	0.828	0.160	0.336
	RF	0.476	0.627	0.955
Radiomics model versus T1V + T1D model	LR	**0.018**	0.653	0.760
	RF	**0.040**	0.633	0.809
Clinical + radiomics model versus clinical + imaging model	LR	< 0.01	0.001	0.917
	RF	< 0.01	< 0.01	1.000
Imaging + radiomics model versus clinical + imaging model	LR	< 0.01	**0.011**	0.690
	RF	< 0.01	< 0.01	1.000
Clinical + radiomics model versus imaging + radiomics model	LR	0.228	0.609	0.838
	RF	0.120	0.445	0.192
Imaging + clinical + radiomics model versus radiomics model	LR	**0.018**	0.424	0.236
	RF	**0.023**	0.526	0.870
Imaging + clinical + radiomics model versus imaging + clinical model	LR	< 0.001	< 0.001	0.886
	RF	< 0.001	< 0.001	0.216
Imaging + clinical + radiomics model versus clinical + radiomics model	LR	**0.010**	0.271	0.072
	RF	**0.025**	0.244	0.978
Imaging + clinical + radiomics model versus imaging + radiomics model	LR	**0.018**	0.347	0.046
	RF	0.233	0.592	0.230

Bold values are statistically significant with *p* < 0.05 corrected by false discovery rate (FDR)

### Performance of fusion models using clinicoradiological and radiomics features

Clinical + radiomics model and imaging + radiomics model achieve similar predictive efficiency in training, validation and test cohorts, but they are better than clinical + imaging model (FDR *p* < 0.05). The combined Ki67 prediction model incorporating clinical, imaging and radiomics model achieves excellent predictive efficiency in training (AUC = 0.904, 95% CI 0.849–0.960), validation (AUC = 0.870, 95% CI 0.775–0.965) and test (AUC = 0.815, 95% CI 0.688–0.941) cohorts. The combined Ki67 prediction model exhibits better than clinical + imaging model (FDR *p* < 0.001), clinical + radiomics model (FDR *p* = 0.010) and imaging + radiomics model (FDR *p* = 0.018) (Tables 3, 4, Fig. 3C–E).

### Evaluation and verification of the combined Ki67 prediction model

**Fig. 4:**
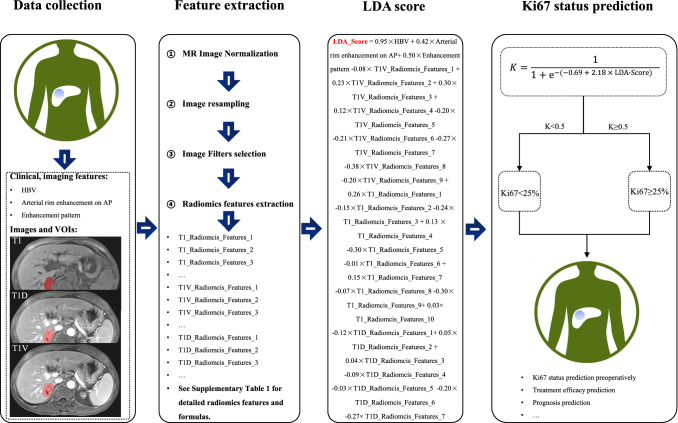
The predictive flowchart of the combined Ki67 prediction model

**Fig. 5:**
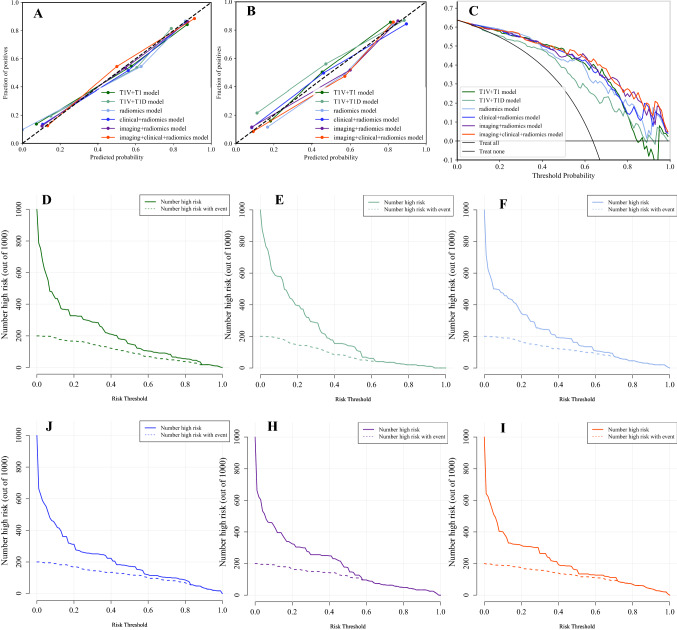
Evaluation and verification of the fusion models. (**A**, **B**) Calibration curves of the fusion models in term of agreement between predicted and actual Ki67 status in the training (**A**) and validation (**B**) cohort. X-axis represents predicted Ki67 status, Y-axis represents actual Ki67 status, and dashed line represents the ideal prediction. (**C**) Decision curves of the fusion models. The grey line represents the assumption that all patients with high Ki67 status, and the horizontal black line represents the assumption that no patient with high Ki67 status. (**D–I**) Clinical impact curves of the fusion models. The dashed line is the actual number of ICC patients with high Ki67 status. The combined Ki67 model has the largest risk threshold range of 0.5–1.0, and the predicted Ki67 status is highly consistent with the actual Ki67 status with risk thresholds ranging between 0.5 and 1.0

**Fig. 6:**
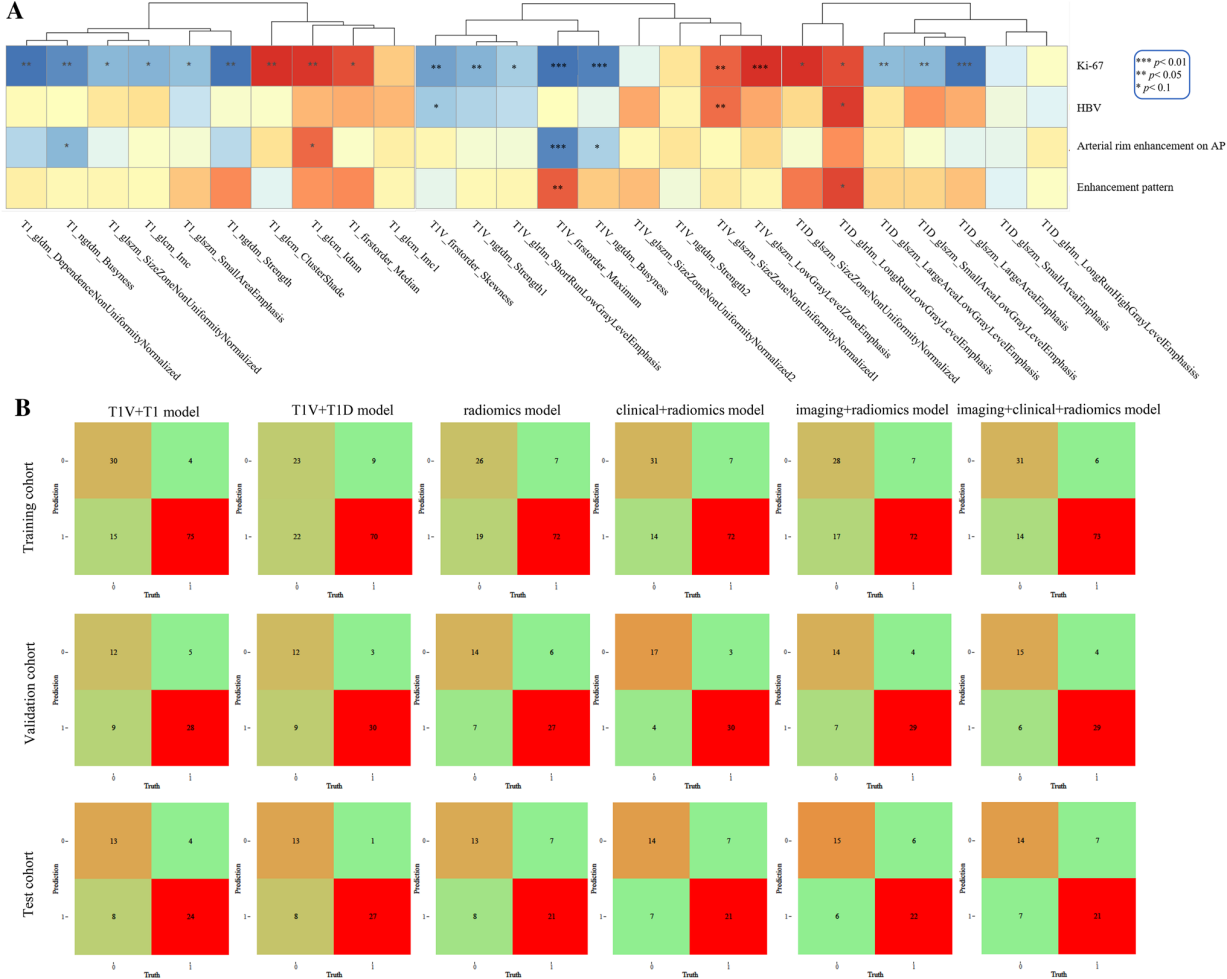
(**A**) The heatmap of the correlation between Ki67 status, clinicoradiological features and radiomics features. (**B**) The confusion matrixes of the fusion models in the training, validation and test models

The flowchart of the combined Ki67 prediction model is shown in Fig. 4. Calibration curves show the goodness of fit between the predicted Ki67 status by using the combined Ki67 prediction model and actual Ki67 status in the training (*p* = 0.787) and validation (*p* = 0.742) cohorts (Fig. 5A, B). Decision curves show that radiomics model, clinical + radiomics, imaging + radiomics model and the combined Ki67 prediction model could obtain net benefit by predicting Ki67 status of all range risk threshold, and the combined Ki67 prediction model exhibits the highest net benefit (Fig. 5C). To further assess the clinical utility of models, clinical impact curves show that the combined Ki67 model has the largest risk threshold range of 0.5–1.0, and the predicted Ki67 status is highly consistent with the actual Ki67 status with risk thresholds ranging between 0.5 and 1.0 (Fig. 5D–I). The heatmap of the correlation between Ki67 status, clinicoradiological features and radiomics features is shown in Fig. 6A and Table S5. The confusion matrixes of the fusion models in the training, validation and test models are shown in Fig. 6B.

## Discussion

In this study, we established a multiparametric model for predicting Ki67 status in ICC patients preoperatively. The final combined Ki67 prediction model incorporates clinical, imaging and radiomics features, and it exhibits an excellent predictive efficiency.

As previous studies [11, 21], we classified ICC lesions into low Ki67 status group and high Ki67 status group by 25% in our study. However, in the majority of studies on predicting Ki67 status of HCC preoperatively, the cut-off value of low and high Ki67 status is usually selected as 10–15% [17, 26]. This may be due to the fact that ICC is a much more aggressive cancer than HCC [27, 28]. Tsokos et al*.* [29] found that well-differentiated ICC had higher Ki67 LI than benign proliferations (22.7% vs. 1.4%, respectively; *p* < 0.001), and none of the benign biliary lesions had Ki67 LI greater than 10%. Therefore, it is inferred that 10% is more likely to be the cut-off value for differentiating benign biliary lesion and ICC, and 25% are more rational in differentiating low and high Ki67 status in ICC lesions.

The multivariate analysis shows HBV is the only independent clinical predictor of Ki67 status in our study. HBV and cirrhosis are risk factors for ICC, with overall odds ratios of 5.10 and 22.92, respectively [30], and Tovoli et al*.* [27] also found that cirrhotic patients with ICCs have different clinical presentation and outcomes. However, Peng et al*.* [31] showed there was no statistical differences between low and high Ki67 status in ICC patients, it may be due to an irrational cut-off value of 10% was selected in his study. The multivariate analysis also shows that arterial rim enhancement on AP and enhancement pattern are two independent imaging predictors of Ki67 status. Min et al*.* [32] found that arterial peripheral rim enhancement pattern was prognostic factor for increased risk of death, which is consistent with the relationship between arterial rim enhancement on AP and Ki67. Our study also suggests that ICC lesions with enhancement pattern like HCC (wash-in and wash-out) may yield a higher Ki67 LI.

Since neither clinical model nor imaging model nor clinical + imaging model can achieve desirable AUC, so we further analyze the predictive performance of radiomics features using 6 single MR sequences. Obviously, none of them can meet the prediction requirements. But the single MR sequence models including T1, T1V and T1D models show stable predictive efficiency, more importantly, pairwise combination models of these three models yield stable predictive efficacy and higher AUCs in validation than training cohort. Thereby, the final radiomics model constructed with T1, T1V and T1D models may be more reliable. Although the final radiomics model performs better than T1V + T1D models in the training cohort, it performs poorly in test cohort and its specificity is unsatisfying, so a fusion model incorporates clinicoradiological and radiomics features is needed. Clinical + radiomics model, imaging + radiomics and the combined Ki67 prediction model achieve better than clinical + imaging model, indicating that radiomics features are vital to the prediction of Ki67 status in ICC patients. Decision curves show that the combined Ki67 model could predict Ki67 status in all range risk threshold and obtain the highest net benefit. Clinical impact curves show that the combined Ki67 model has the largest risk threshold range of 0.5–1.0 to identify the Ki67 status accurately.

The principle of the LASSO algorithm is to compress coefficients of features by introducing a regularization parameter, and remove some features with zero coefficients, so as to achieve the purpose of feature selection [33]. Rad-score is obtained by matrix multiplication of the features and their coefficients obtained by the LASSO algorithm, usually as an independent prediction signature. However, Rad-score completely relies on the feature selection results of LASSO, and the category information is not directly correlated. LDA is a supervised feature dimensionality reduction method, which takes the category information studied as a priori knowledge [34]. The main purpose of LDA was to maximize the variation between samples of different categories and further fit a combined signature that was more suitable for discriminating high and low Ki67 status of ICCs in our study. Therefore, the combined signature obtained by the LDA method, with maximum inter-class variance and minimum intra-class variance, may could make the samples to be predicted obtain the best separability.

Recently, there have been several studies on the Ki67 prediction in HCC based on CT [26] and MR [17, 35], but there is no study on the Ki67 prediction in ICC based on CT or MR. In our study, a total of 22 radiomics features (T1WI image: 10, PVP image: 9, DP image: 7) are correlated with the Ki67 status (Fig. 6A and Table S5). And T1V_firstorder_Maximum (*r* = − 0.225, *p* = 0.002), T1V_glszm_LowGrayLevelZoneEmphasis (*r* = 0.216, *p* = 0.004), T1V_ngtdm_Busyness (*r* = − 0.210, *p* = 0.005), and T1D_glszm_LargeAreaEmphasis (*r* = − 0.212, *p* = 0.005) have strong correlation, which happens to explain why T1V model shows the most sable predictive efficiency among single MR sequence models. Peng et al*.* [31] predicted Ki67 status in ICC based on ultrasound radiomics features, principal component analysis as an unsupervised feature dimensionality reduction method, was used before LASSO in the feature selection, therefore, the features finally used to predict Ki67 status were weakly interpretable. The study about Ki67 status prediction in HCC conducted by Ye et al*.* [17] was similar to our study, only two glrlm features (LongRunHighGrayLevelEmphasis, LongRunLowGrayLevelEmphasis) are identical to our study, suggesting that these two features may be independently related to the Ki67 status, regardless of tumor type.

There are several limitations in our study. Firstly, we define “Ki67 LI ≥ 25%” as high Ki67 status in our study according to previous studies, however, the reason why the Ki67 cut-off value of ICC is higher than that of HCC needs further study. Secondly, selection bias is inevitable in retrospective study, and an estimation of the effect of the splitting procedure may be needed in future study. Thirdly, there is no study on the Ki67 prediction in ICC based on MR in the past, thus more studies in this area are needed to compare and verify our study, and the correlation between radiomics features and Ki67 status, complex clinicoradiological features need to be further explained. Finally and importantly, larger cohorts from other centers are needed to be enrolled for prospective validation of our Ki67 prediction model.

In summary, the combined Ki67 model incorporating clinical feature (HBV), imaging features (arterial rim enhancement on AP and enhancement pattern) and radiomics features (on T1, T1V and T1D sequences) is a potential biomarker in Ki67 prediction, and the flowchart of the combined Ki67 prediction model may be a potential tool in Ki67 stratification of ICC patients (Fig. 4).

## Supplementary Information

Below is the link to the electronic supplementary material.

Supplementary file1 (PDF 398 KB)

Supplementary file2 (PDF 441 KB)

Supplementary file3 (PDF 259 KB)

Supplementary file4 (DOCX 1645 KB)
