# Supplementary material for: Development and validation of combined Ki67 status prediction model for intrahepatic cholangiocarcinoma based on clinicoradiological features and MRI radiomics
Source: Radiol Med. 2023 Feb 11;128(3):274–88. doi: 10.1007/s11547-023-01597-7 (PMC10020304; doi:10.1007/s11547-023-01597-7)
Supplement: Supplementary file 1 — Supplementary file1 (PDF 398 KB) [file 11547_2023_1597_MOESM1_ESM.pdf]

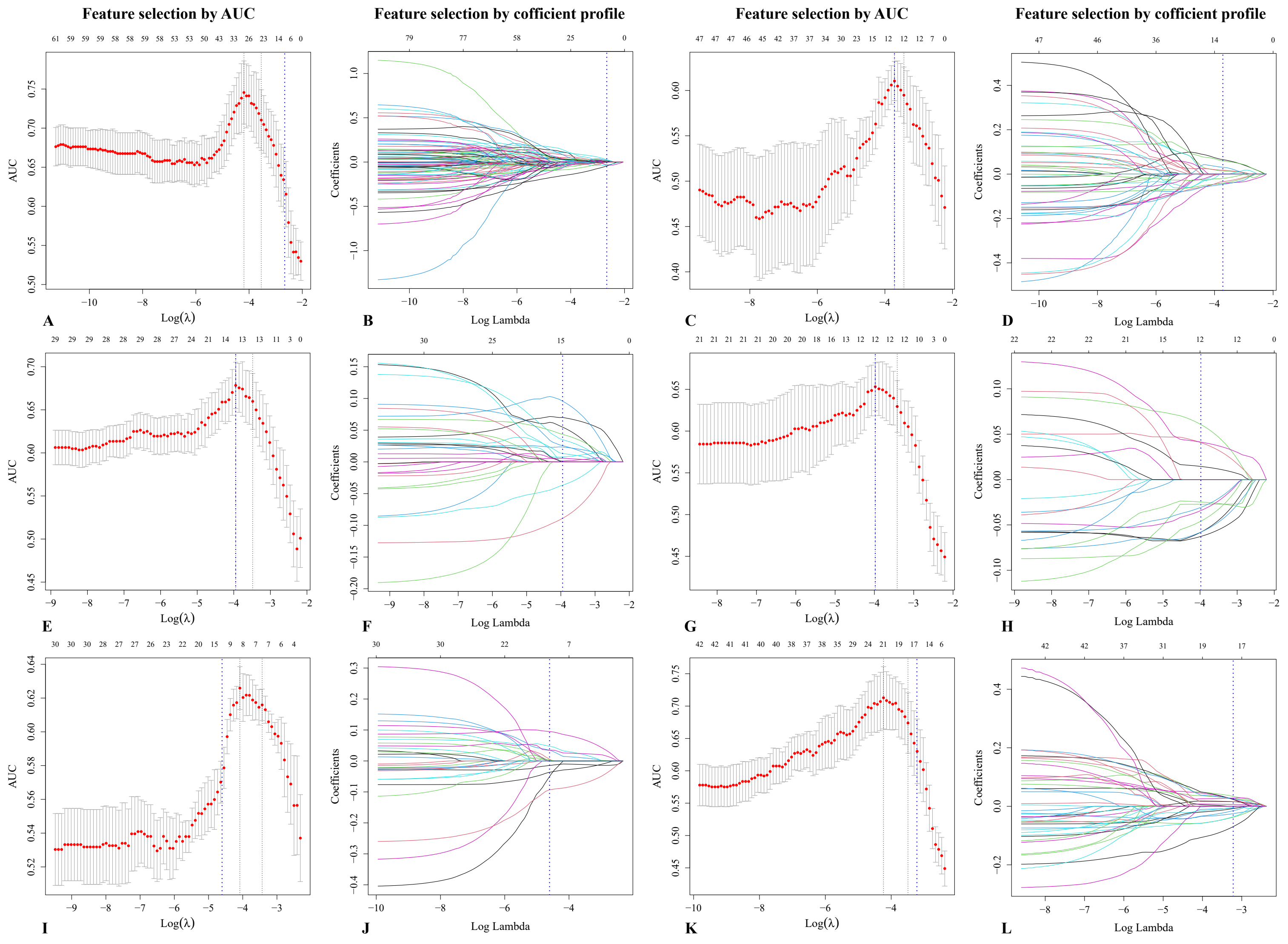
Feature selection by AUC
Feature selection by cofficient profile
Feature selection by AUC
Feature selection by cofficient profile
AUC
Log(λ)
A
Coefficients
Log Lambda
B
AUC
Log(λ)
C
Coefficients
Log Lambda
D
AUC
Log(λ)
E
Coefficients
Log Lambda
F
AUC
Log(λ)
G
Coefficients
Log Lambda
H
AUC
Log(λ)
I
Coefficients
Log Lambda
J
AUC
Log(λ)
K
Coefficients
Log Lambda
L
