# Supplementary figures and images for: Development and validation of combined Ki67 status prediction model for intrahepatic cholangiocarcinoma based on clinicoradiological features and MRI radiomics
Source: Radiol Med. 2023 Feb 11;128(3):274–88. doi: 10.1007/s11547-023-01597-7 (PMC10020304; doi:10.1007/s11547-023-01597-7)

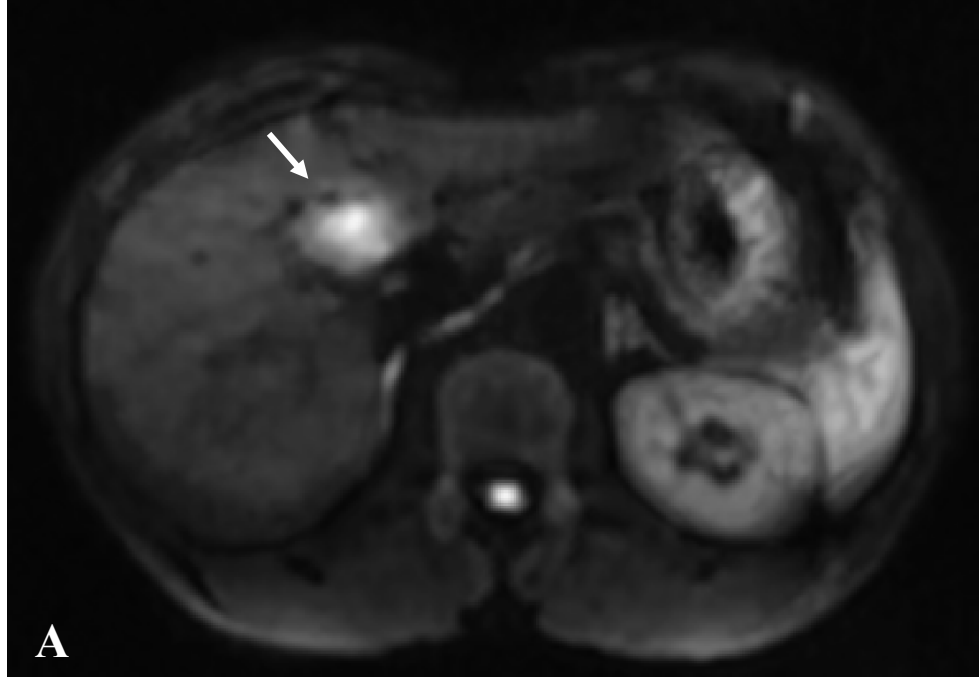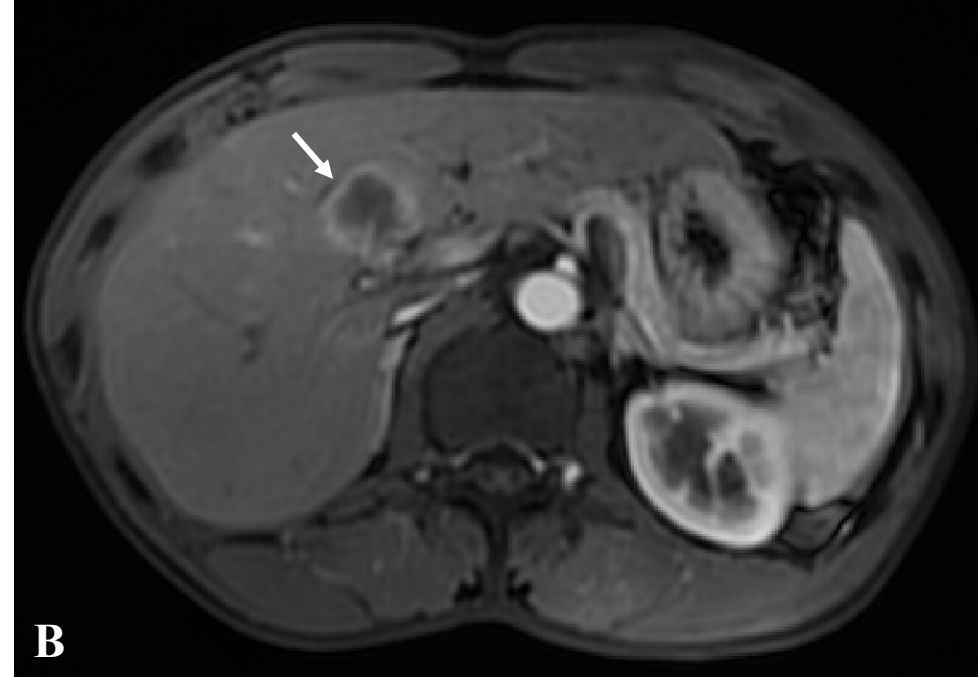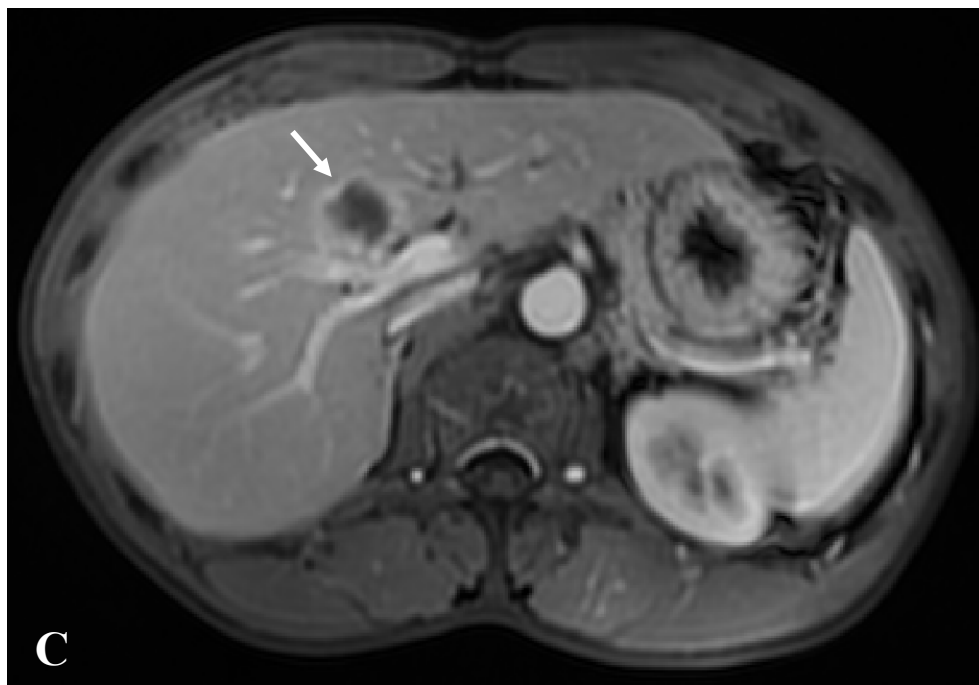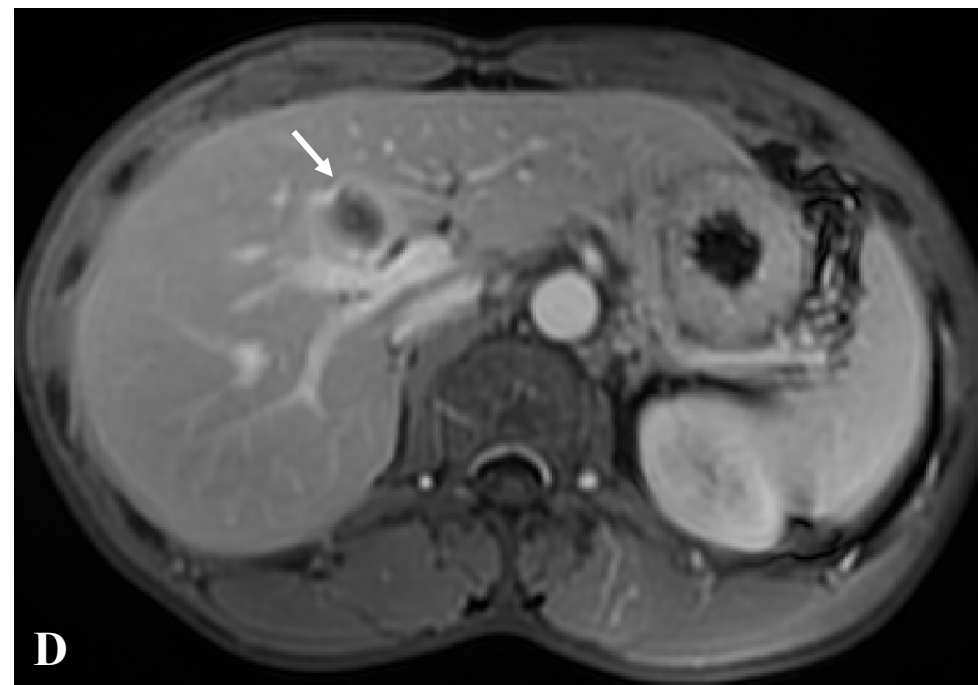

Supplement: Supplementary file 2 — Supplementary file2 (PDF 441 KB) [file 11547_2023_1597_MOESM2_ESM.pdf]

**Training cohort**

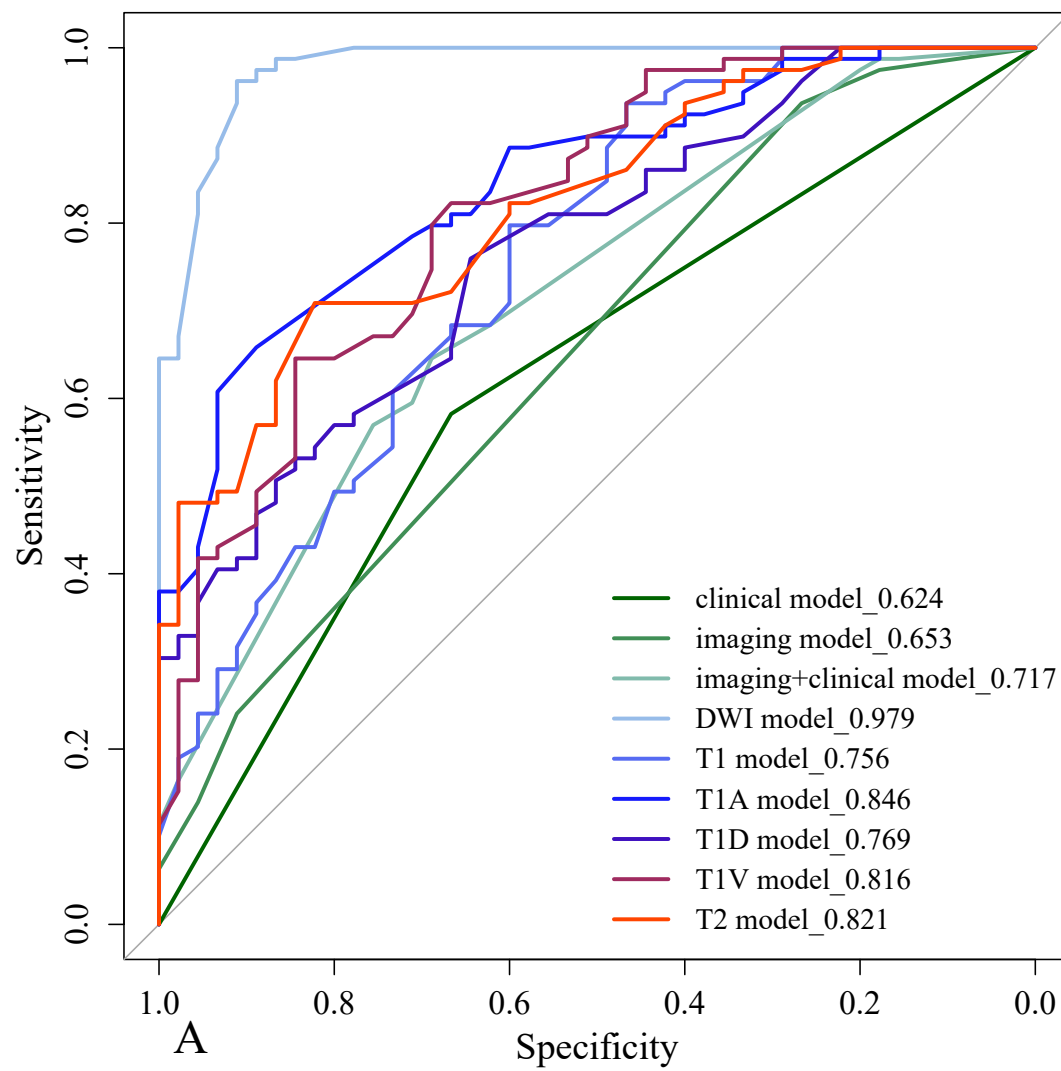

**Validation cohort**

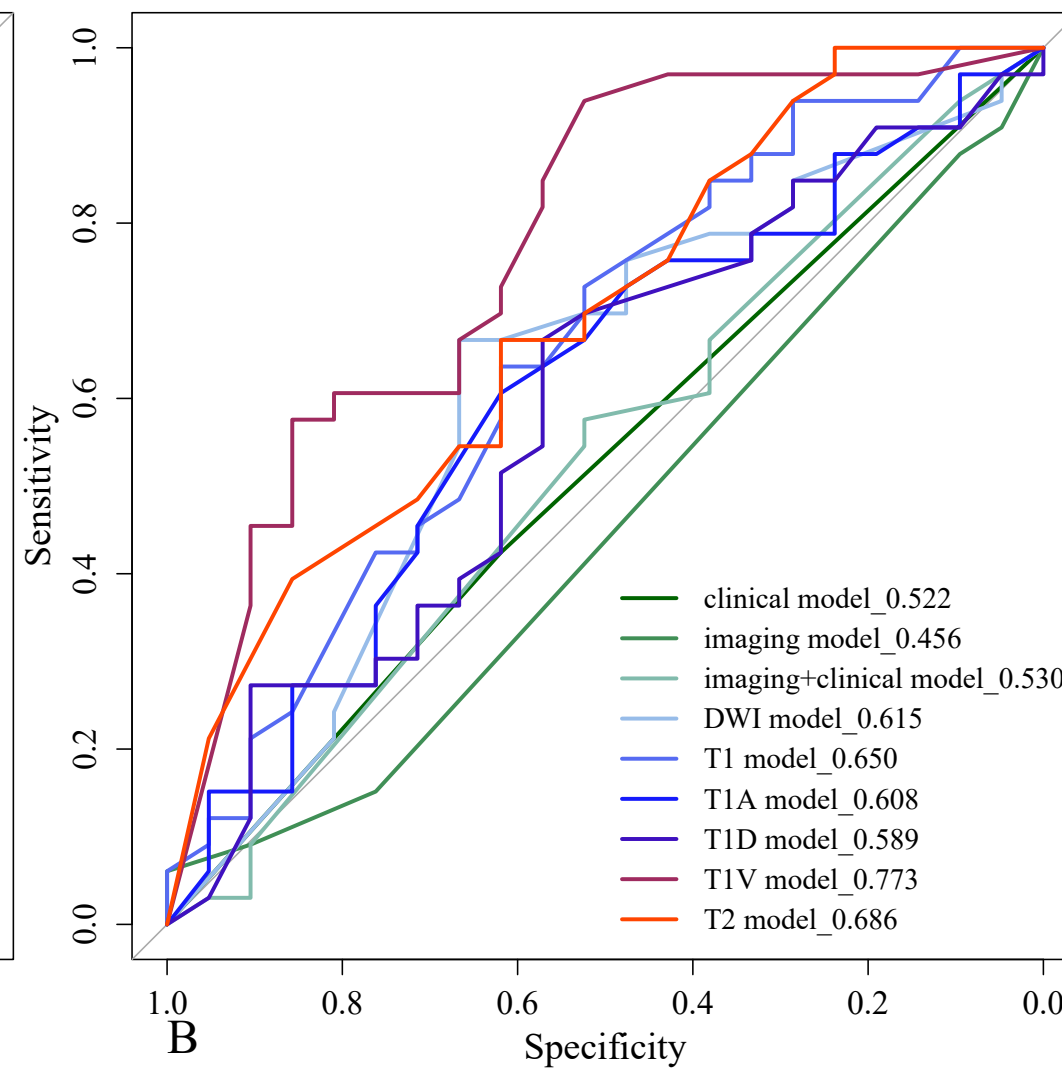

**Test cohort**

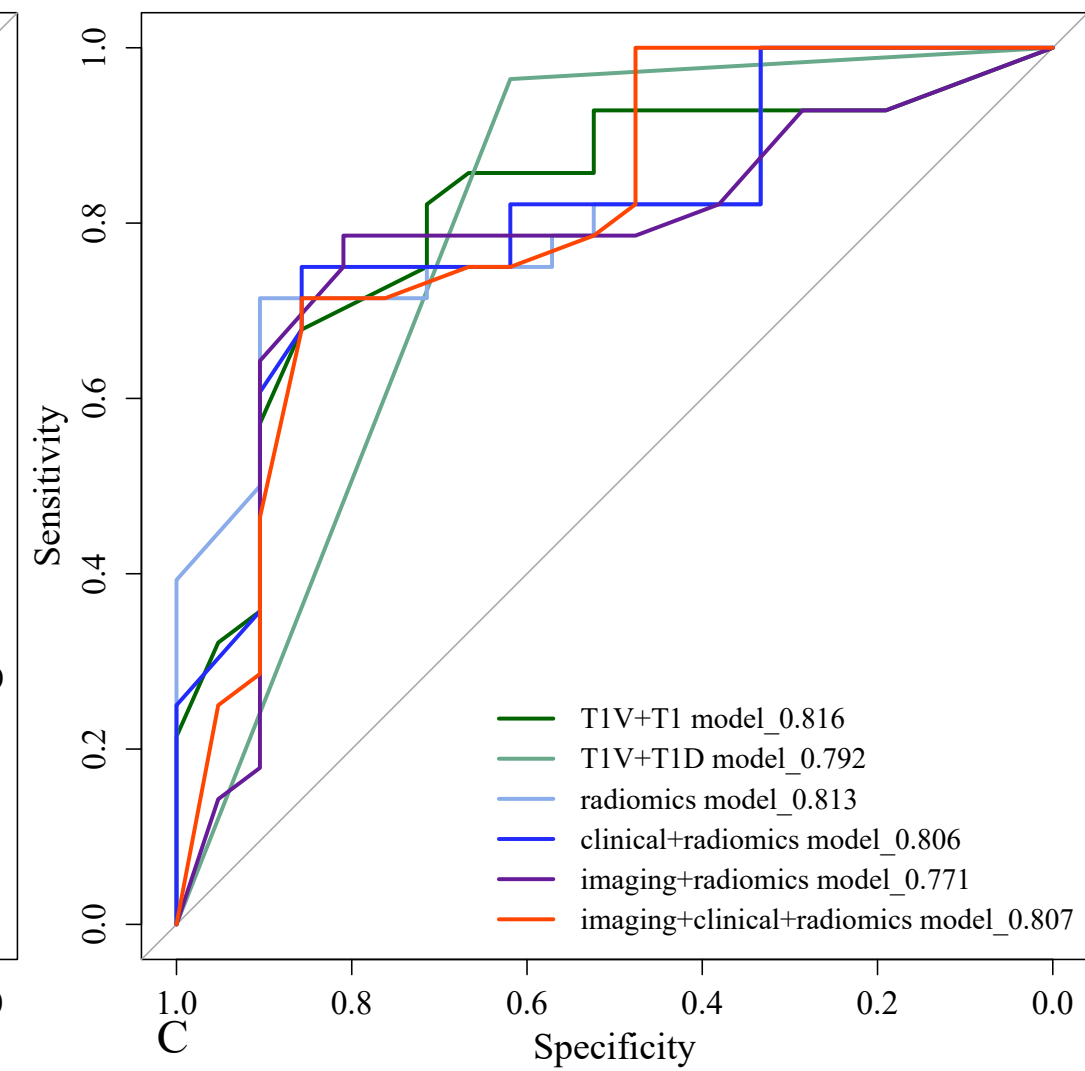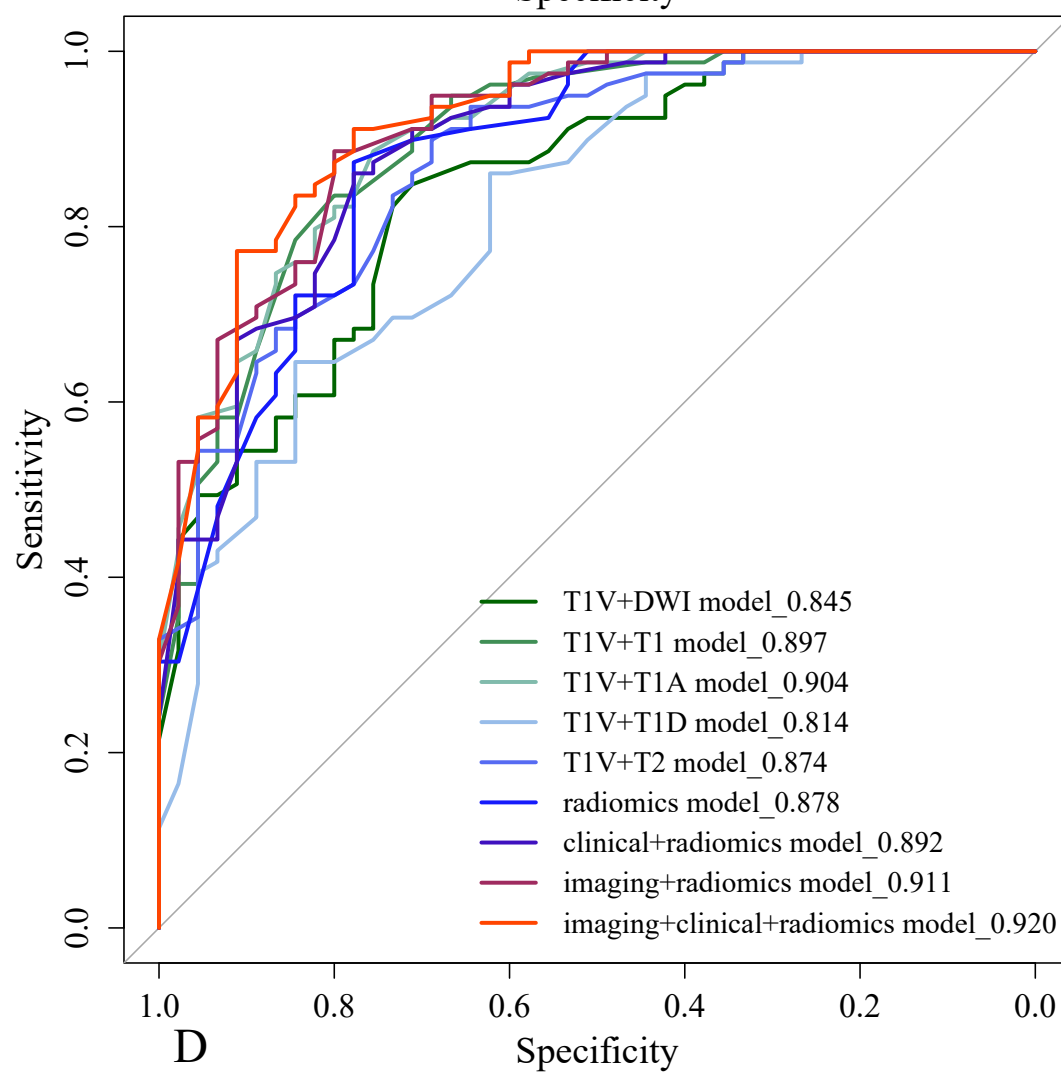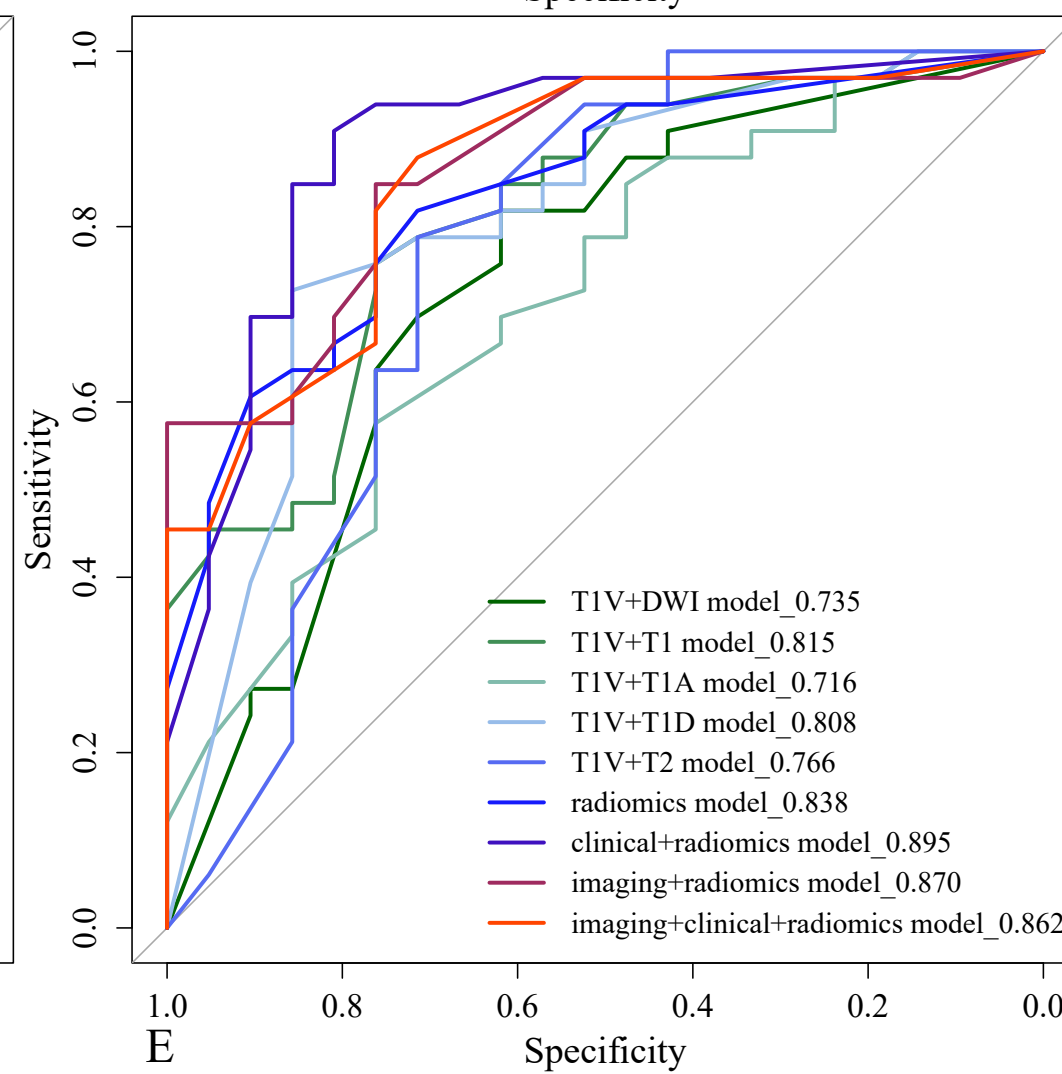

Supplement: Supplementary file 3 — Supplementary file3 (PDF 259 KB) [file 11547_2023_1597_MOESM3_ESM.pdf]
