# Supplementary material for: Development and validation of combined Ki67 status prediction model for intrahepatic cholangiocarcinoma based on clinicoradiological features and MRI radiomics
Source: Radiol Med. 2023 Feb 11;128(3):274–88. doi: 10.1007/s11547-023-01597-7 (PMC10020304; doi:10.1007/s11547-023-01597-7)
Supplement: Supplementary file 4 — Supplementary file4 (DOCX 1645 KB) [file 11547_2023_1597_MOESM4_ESM.docx]

**Supplementary data**

**Supplemental Table 1.** Baseline clinicoradiological features of ICC patients in test cohort.

| Features | Test cohort (*n*=49) | | |
| --- | --- | --- | --- |
|  | Ki67<25% (*n*=21) | Ki67≥25% (*n*=28) | *p*-Intra |
| *Clinical features* | | | |
| Age (years)^a^ | 64 (11.218) | 64 (9.446) | 0.818 |
| Gender |  |  | 0.788 |
| Male | 6 (28.6) | 9 (32.1) |  |
| Female | 15 (71.4) | 19 (67.9) |  |
| HBV |  |  | 1.000 |
| Negative | 12 (57.1) | 16 (57.1) |  |
| Positive | 9 (42.9) | 12 (42.9) |  |
| AFP |  |  | 0.500 |
| <20 ng/ml | 21 (100.0) | 26 (92.9) |  |
| ≥20 ng/ml | 0 (0.0) | 2 (7.1) |  |
| CEA |  |  | 1.000 |
| <5 ng/ml | 18 (85.7) | 24 (85.7) |  |
| ≥5 ng/ml | 3 (14.3) | 4 (14.3) |  |
| CA199 |  |  | 0.560 |
| <34 U/ml | 11 (52.4) | 17 (60.7) |  |
| ≥34 U/ml | 10 (47.6) | 11 (39.3) |  |
| Edmondson-Steiner grade |  |  | 0.000 |
| I-II | 20 (95.2) | 11 (39.3) |  |
| II-IV | 1 (4.8) | 17 (60.7) |  |
| *MR imaging features* | | | |
| Tumor size(mm)^b^ | 37.10 (29.60-42.20) | 42.80 (36.88-56.45) | 0.022 |
| Tumor morphology |  |  | 0.762 |
| (Hemi-)spherical and oval | 11 (52.4) | 8 (28.6) |  |
| Lobulated | 8 (38.1) | 16 (57.1) |  |
| Irregular | 2 (9.5) | 4 (14.3) |  |
| SI on T1WI |  |  | 0.317 |
| Low | 21 (100.0) | 28 (100.0) |  |
| Moderate | 0 (0) | 0 (0) |  |
| High | 0 (0) | 0 (0) |  |
| SI on T2WI-FS |  |  | 0.317 |
| Low | 0 (0) | 0 (0) |  |
| Moderate | 0 (0) | 0 (0) |  |
| High | 21 (100.0) | 28 (100.0) |  |
| Target sign on T2WI-FS |  |  | 0.560 |
| Negative | 11 (52.4) | 17 (60.7) |  |
| Positive | 10 (47.6) | 11 (39.3) |  |
| Target sign on DWI |  |  | 0.458 |
| Negative | 12 (57.1) | 13 (46.4) |  |
| Positive | 9 (42.9) | 15 (53.6) |  |
| Intrahepatic duct dilatation |  |  | 0.047 |
| Negative | 15 (71.4) | 12 (42.9) |  |
| Positive | 6 (28.6) | 16 (57.1) |  |
| Hepatic capsular retraction |  |  | 0.924 |
| Negative | 16 (76.2) | 21 (75.0) |  |
| Positive | 5 (23.8) | 7 (25.0) |  |
| Visible vessel penetration |  |  | 0.271 |
| Negative | 11 (52.4) | 19 (67.9) |  |
| Positive | 10 (47.6) | 9 (32.1) |  |
| Peripheral hepatic enhancement |  |  | 0.934 |
| Negative | 10 (47.6) | 13 (46.4) |  |
| Positive | 11 (52.4) | 15 (53.6) |  |
| Arterial rim enhancement on AP |  |  | 0.350 |
| Negative | 7 (33.3) | 6 (21.4) |  |
| Positive | 14 (66.7) | 22 (78.6) |  |
| Complete rim on AP |  |  | 0.311 |
| Negative | 4 (28.6) | 10 (45.5) |  |
| Positive | 10 (71.4) | 12 (54.5) |  |
| Enhancement pattern |  |  | 0.952 |
| Gradual and filling | 20 (95.2) | 20 (71.4) |  |
| Arterial and persistent | 1 (4.8) | 5 (17.9) |  |
| Wash-in and wash-out | 0 (0.0) | 3 (10.7) |  |
| LI-RADS |  |  | 0.632 |
| LR-3 | 0 (0.0) | 0 (0.0) |  |
| LR-4 | 0 (0.0) | 0 (0.0) |  |
| LR-5 | 5 (23.8) | 4 (14.3) |  |
| LR-M | 16 (76.2) | 24 (85.7) |  |
| LR-TIV | 0 (0.0) | 0 (0.0) |  |

Unless otherwise stated, data are shown as number of patients with percentage in parentheses.

^a^ Data are means with standard deviation in parentheses.

^b^ Data are medians with interquartile ranges in parentheses.

**Supplemental Table 2.** Gd-DTPA MR imaging sequences and parameters of 3.0T uMR 770 scanner.

| Parameter | T2WI-FS | DWI | IP-OP T1WI | quick3d T1WI |
| --- | --- | --- | --- | --- |
| Repetition time (msec) | 2000 | 4165 | 4.27 | 3.3 |
| Echo time (msec) | 106.2 | 66.3 | 2.5 and 1.21 | 1.5 |
| Section thickness (mm) | 6-7 | 6-7 | 3-4 | 3 |
| Matrix size | 256×256 | 128×101 | 288×168 | 320×216 |
| Field of view (mm^2^) | 346×346 | 300×300 | 300×300 | 270×270 |
| Gap (mm) | 1.8-2.1 | 1.8-2.1 | 0 | 0 |
| Flip Angle (°) | 80 | 90 | 10 | 10 |
| Average | 1 | 5 | 1 | 1 |
| FS scheme | Frequency-selective Fat Suppression | / | / | Frequency-selective Fat Suppression |
| MR coil | Body Array Coil - 12 | | | |

**Supplemental Table 3.** The feature numbers of each single MR sequence during the procedure of feature selection.

| Sequences | Input features | Test-Retest (≥0.75) | Inter-observer (≥0.75) | Test-Retest+ Inter-observer (≥0.75) | Correlation analysis | Multiple synteny analysis | LASSO selection |
| --- | --- | --- | --- | --- | --- | --- | --- |
| DWI | 2600 | 2579 | 2566 | 2565 | 521 | 80 | 12 |
| T1 | 2600 | 2578 | 2560 | 2555 | 158 | 47 | 11 |
| T1A | 2600 | 2581 | 2581 | 2571 | 50 | 30 | 13 |
| T1V | 2600 | 2566 | 2567 | 2560 | 43 | 22 | 12 |
| T1D | 2600 | 2581 | 2572 | 2571 | 106 | 30 | 11 |
| T2 | 2600 | 2579 | 2574 | 2570 | 213 | 42 | 17 |

**Supplemental Table 4.** The detailed information of radiomics features in each single MR sequence.

| Sequences | Radiomics types | Filters | Radiomics Features | Lasso Coefficients |
| --- | --- | --- | --- | --- |
| DWI | glcm | log.sigma.0.5.mm.3d | imc1 | 0.140 |
| DWI | glcm | wavelet.hll | wavelet.hll.imc 2 | 0.123 |
| DWI | gldm | log.sigma.1.5.mm.3d | dependence variance | 0.097 |
| DWI | first order | wavelet.lhh. | skewness | 0.091 |
| DWI | glcm | Laplacian sharpening | cluster shade | 0.081 |
| DWI | glcm | wavelet.lhl | imc1 | 0.052 |
| DWI | glcm | Laplacian sharpening | inverse variance | -0.052 |
| DWI | glcm | wavelet.llh | correlation | -0.054 |
| DWI | first order | wavelet.hhl | skewness | -0.087 |
| DWI | glszm | wavelet.hhl | size zone nonuniformity normalized | -0.087 |
| DWI | glszm | wavelet.llh | small area emphasis | -0.129 |
| DWI | glszm | log.sigma.0.5.mm.3d | size zone nonuniformity normalized | -0.141 |
| T1 | first order | wavelet.hhl | median | 0.365 |
| T1 | glcm | box sigma image | idmn | 0.268 |
| T1 | glszm | shot noise | low gray level zone emphasis | 0.176 |
| T1 | glcm | wavelet.hlh | cluster shade | 0.080 |
| T1 | glcm | shot noise | imc1 | 0.068 |
| T1 | glcm | wavelet.llh | imc2 | -0.105 |
| T1 | glszm | wavelet.hhh | size zone nonuniformity normalized | 0.005 |
| T1 | glszm | log.sigma.1.5.mm.3d | small area emphasis | -0.180 |
| T1 | ngtdm | wavelet.hlh | strength | -0.183 |
| T1 | gldm | recursive gaussian | dependence nonuniformity normalized | -0.212 |
| T1 | ngtdm | Speckle noise | busyness | -0.187 |
| T1A | gldm | log.sigma.1.0.mm.3d | large dependence high gray level emphasis | 0.094 |
| T1A | glszm | wavelet.lhh | gray level nonuniformity normalized | 0.068 |
| T1A | ngtdm | wavelet.hlh | contrast | 0.053 |
| T1A | glcm | wavelet.hlh | cluster shade | 0.039 |
| T1A | glszm | Laplacian sharpening | gray level nonuniformity | 0.031 |
| T1A | glcm | normalize | correlation | 0.027 |
| T1A | gldm | wavelet.hll | large dependence low gray level emphasis | 0.020 |
| T1A | glcm | box mean | idmn | 0.016 |
| T1A | first order | wavelet.llh | median | 0.012 |
| T1A | first order | wavelet.llh | mean | 0.002 |
| T1A | first order | normalize | maximum | 0.000 |
| T1A | gldm | wavelet.lll | small dependence low gray level emphasis | -0.018 |
| T1A | glrlm | wavelet.hll | long run high gray level emphasis | -0.082 |
| T1D | glrlm | wavelet.hlh | long run low gray level emphasis | 0.071 |
| T1D | first order | wavelet.hlh | median | 0.045 |
| T1D | gldm | Laplacian sharpening | small dependence low gray level emphasis | 0.024 |
| T1D | glszm | log.sigma.1.5.mm.3d | size zone nonuniformity normalized | 0.009 |
| T1D | gldm | additive gaussian noise | large dependence low gray level emphasis | 0.001 |
| T1D | glszm | log.sigma.0.5.mm.3d | gray level nonuniformity normalized | 0.001 |
| T1D | glrlm | wavelet.hll | long run high gray level emphasis | -0.005 |
| T1D | glszm | wavelet.hhl | small area emphasis | -0.014 |
| T1D | glszm | wavelet.lll | small area emphasis | -0.022 |
| T1D | glszm | wavelet.hhh | small area low gray level emphasis | -0.032 |
| T1D | glszm | log.sigma.1.5.mm.3d | large area low gray level emphasis | -0.049 |
| T1V | glszm | log.sigma.0.5.mm.3d | size zone nonuniformity normalized | 0.063 |
| T1V | glszm | shot noise | size zone nonuniformity normalized | 0.042 |
| T1V | glszm | wavelet.hlh | low gray level zone emphasis | 0.042 |
| T1V | glcm | normalize | idmn | 0.016 |
| T1V | first order | normalize | maximum | -0.024 |
| T1V | glszm | normalize | gray level nonuniformity | -0.026 |
| T1V | ngtdm | wavelet.hhl | strength | -0.034 |
| T1V | gldm | box sigma image | dependence nonuniformity | -0.038 |
| T1V | ngtdm | Laplacian sharpening | busyness | -0.045 |
| T1V | glrlm | normalize | short run low gray level emphasis | -0.061 |
| T1V | first order | log.sigma.2.mm.3d | skewness | -0.062 |
| T1V | ngtdm | wavelet.hlh | strength | -0.062 |
| T2 | glcm | speckle noise | correlation | 0.126 |
| T2 | first order | wavelet.llh | kurtosis | 0.120 |
| T2 | glcm | wavelet.hlh | inverse variance | 0.066 |
| T2 | first order | wavelet.lhh | mean | 0.066 |
| T2 | gldm | box sigma image | dependence variance | 0.113 |
| T2 | glcm | recursive gaussian | correlation | 0.116 |
| T2 | glcm | binomial blur image | correlation | 0.113 |
| T2 | gldm | normalize | dependence entropy | 0.032 |
| T2 | glcm | wavelet.hlh | correlation | -0.014 |
| T2 | gldm | log.sigma.1.5.mm.3d | large dependence high gray level emphasis | -0.103 |
| T2 | glcm | box mean | imc1 | -0.043 |
| T2 | gldm | wavelet.hll | small dependence low gray level emphasis | -0.019 |
| T2 | gldm | wavelet.hhl | large dependence high gray level emphasis | -0.103 |
| T2 | first order | log.sigma.0.5.mm.3d | kurtosis | -0.075 |
| T2 | glrlm | wavelet.lhl | long run low gray level emphasis | -0.159 |
| T2 | first order | wavelet.hll | skewness | -0.160 |
| T2 | first order | log.sigma.2.mm.3d | kurtosis | -0.310 |

Details and formulas of features:

https://pyradiomics.readthedocs.io/en/latest/features.html#module-radiomics.firstorder

$$K=\frac{1}{1+e^{-(-0.69 + 2.18 \times\boldsymbol{LDA\_}\boldsymbol{S}\mathbf{core})}}$$

LDA_Score = 0.95 × HBV + 0.42 × Arterial rim enhancement on AP + 0.5 × Enhancement pattern - 0.08 × T1V wavelet.HLH ngtdm Strength + 0.23 × T1V shot noise glszm Size Zone Nonuniformity Normalized + 0.3 × T1V log.sigma.0.5.mm.3d glszm Size Zone Nonuniformity Normalized + 0.12 × T1V wavelet.HLH glszm Low Gray Level Zone Emphasis - 0.2 × T1V normalize glrlm Short Run Low Gray Level Emphasis - 0.21 × T1V log first order log.sigma.2.mm.3d Skewness - 0.27 × T1V normalize firs order Maximum - 0.38 × T1V wavelet.HHL ngtdm Strength - 0.2 × T1V Laplacian sharpening ngtdm Busyness + 0.26 × T1 wavelet.HHL first order Median - 0.15 × T1 log.sigma.1.5.mm.3d glszm Small Area Emphasis - 0.24 × T1 wavelet.HHH glszm Size Zone Nonuniformity Normalized + 0.13 × T1 wavelet.HLH glcm Cluster Shade - 0.3 × T1 wavelet.HLH ngtdm Strength - 0.01 × T1 shot noise glcm Imc1 + 0.15 × T1 box sigma image glcm Idmn - 0.07 × T1 wavelet.LLH glcm Imc2 - 0.3 × T1 recursive gaussian gldm Dependence Nonuniformity Normalized + 0.03 × T1 speckigtdm Busyness - 0.12 × T1D wavelet.HHH glszm Small Area Low Gray Level Emphasis + 0.05 × T1D wavelet.HHL glszm Small Area Emphasis + 0.04 × T1D log.sigma.1.5.mm.3d glszm Size Zone Nonuniformity Normalized - 0.09 × T1D wavelet.HLL glrlm Long Run High Gray Level Emphasis - 0.03 × T1D wavelet.HLH glrlm Long Run Low Gray Level Emphasis - 0.2 × T1D log.sigma.1.5.mm.3d glszm Large Area Low Gray Level Emphasis - 0.27 × T1D wavelet.LLL glszm Large Area Emphasis

**Supplemental Table 5.** The correlation between Ki67 status, clinicoradiological features and radiomics features.

| Radiomics features | Correlation coefficient | | | | *p* value | | | |
| --- | --- | --- | --- | --- | --- | --- | --- | --- |
|  | Ki67 | HBV | Arterial rim enhancement on AP | Enhancement pattern | Ki67 | HBV | Arterial rim enhancement on AP | Enhancement pattern |
| T1V_ngtdm_Strength1 | -0.164 | -0.121 | -0.028 | 0.009 | **0.029** | 0.107 | 0.706 | 0.907 |
| T1V_glszm_SizeZoneNonUniformityNormalized1 | 0.174 | 0.166 | 0.008 | 0.048 | **0.020** | 0.026 | 0.919 | 0.525 |
| T1V_glszm_SizeZoneNonUniformityNormalized2 | 0.054 | 0.017 | 0.056 | -0.037 | 0.473 | 0.817 | 0.457 | 0.620 |
| T1V_glszm_LowGrayLevelZoneEmphasis | 0.216 | 0.119 | 0.071 | 0.014 | **0.004** | 0.114 | 0.349 | 0.853 |
| T1V_glrlm_ShortRunLowGrayLevelEmphasis | -0.130 | -0.109 | -0.033 | 0.017 | **0.083** | 0.149 | 0.665 | 0.820 |
| T1V_firstorder_Skewness | -0.177 | -0.135 | 0.017 | -0.062 | **0.018** | 0.073 | 0.825 | 0.413 |
| T1V_firstorder_Maximum | -0.225 | -0.004 | -0.205 | 0.178 | **0.002** | 0.962 | 0.006 | 0.017 |
| T1V_ngtdm_Strength2 | -0.065 | 0.116 | 0.010 | 0.099 | 0.388 | 0.123 | 0.892 | 0.191 |
| T1V_ngtdm_Busyness | -0.210 | -0.061 | -0.125 | 0.086 | **0.005** | 0.419 | 0.096 | 0.254 |
| T1_firstorder_Median | 0.147 | 0.083 | -0.018 | 0.109 | **0.050** | 0.270 | 0.814 | 0.148 |
| T1_glszm_SmallAreaEmphasis | -0.126 | -0.086 | -0.029 | 0.068 | **0.093** | 0.256 | 0.696 | 0.370 |
| T1_glszm_SizeZoneNonUniformityNormalized | -0.131 | 0.033 | -0.058 | 0.007 | **0.081** | 0.658 | 0.444 | 0.931 |
| T1_glcm_ClusterShade | 0.168 | -0.017 | 0.042 | -0.062 | **0.025** | 0.824 | 0.573 | 0.408 |
| T1_ngtdm_Strength | -0.184 | -0.023 | -0.097 | 0.109 | **0.014** | 0.760 | 0.197 | 0.146 |
| T1_glcm_Imc1 | 0.062 | 0.076 | 0.020 | 0.006 | 0.411 | 0.311 | 0.790 | 0.937 |
| T1_glcm_Idmn | 0.164 | 0.078 | 0.129 | 0.103 | **0.029** | 0.301 | 0.086 | 0.170 |
| T1_glcm_Imc | -0.136 | 0.044 | -0.018 | -0.001 | **0.070** | 0.559 | 0.806 | 0.987 |
| T1_gldm_DependenceNonUniformityNormalized | -0.187 | 0.000 | -0.101 | 0.020 | **0.012** | 0.999 | 0.179 | 0.788 |
| T1_ngtdm_Busyness | -0.167 | -0.006 | -0.131 | 0.002 | **0.026** | 0.932 | 0.081 | 0.980 |
| T1D_glszm_SmallAreaLowGrayLevelEmphasis | -0.157 | 0.080 | -0.046 | 0.035 | **0.036** | 0.286 | 0.538 | 0.645 |
| T1D_glszm_SmallAreaEmphasis | -0.096 | -0.064 | -0.077 | -0.090 | 0.200 | 0.396 | 0.309 | 0.232 |
| T1D_glszm_SizeZoneNonUniformityNormalized | 0.146 | 0.029 | -0.049 | 0.097 | **0.052** | 0.698 | 0.520 | 0.198 |
| T1D_glrlm_LongRunHighGrayLevelEmphasis | -0.038 | -0.089 | -0.007 | -0.037 | 0.617 | 0.239 | 0.921 | 0.621 |
| T1D_glrlm_LongRunLowGrayLevelEmphasis | 0.126 | 0.137 | 0.082 | 0.128 | **0.094** | 0.067 | 0.277 | 0.089 |
| T1D_glszm_LargeAreaLowGrayLevelEmphasis | -0.153 | 0.013 | 0.004 | 0.033 | **0.042** | 0.863 | 0.960 | 0.666 |
| T1D_glszm_LargeAreaEmphasis | -0.212 | 0.061 | -0.079 | 0.049 | **0.005** | 0.420 | 0.292 | 0.518 |

**
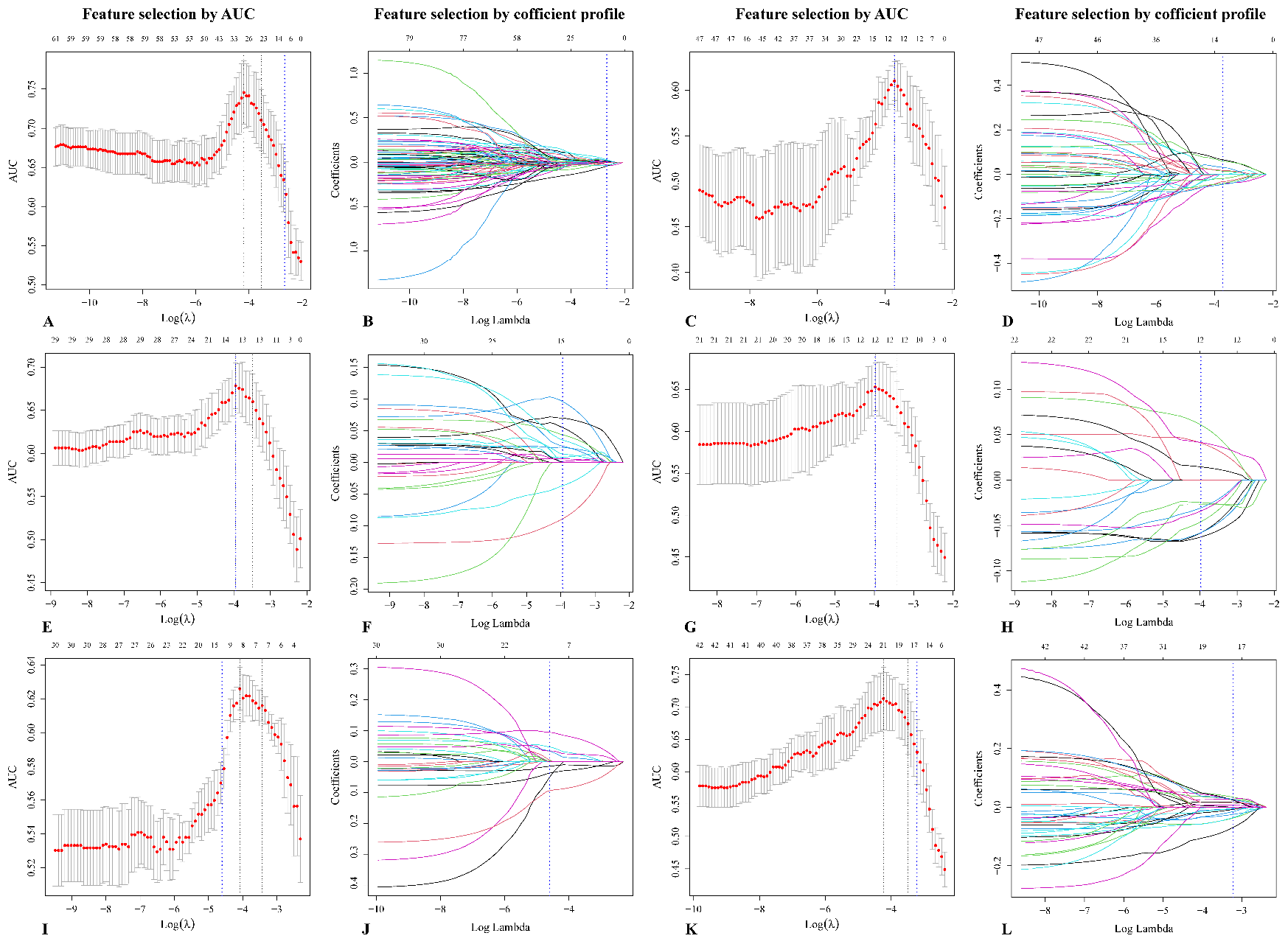
Supplemental Figure 1.** Radiomics feature selection using the least absolute shrinkage and selection operator (LASSO) regression model on diffusion-weighted image (**A, B**), pre-contrast T1-weighted image (**C, D**), arterial phase image (**E, F**), portal vein phase image (**G, H**), delayed phase image (**I, J**) and T2-weighted image (**K, L**).


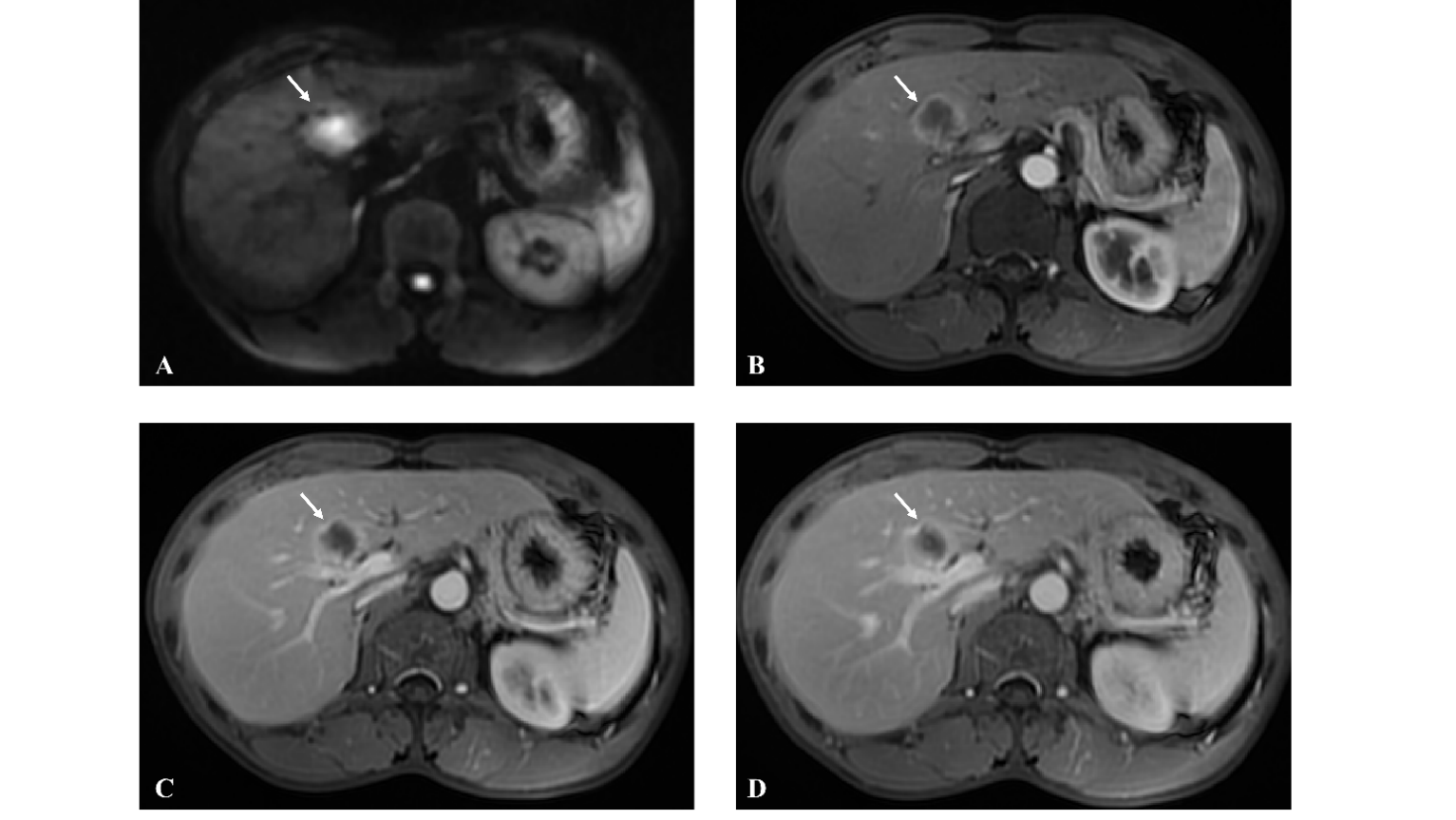


**Supplemental Figure 2.** Example of representative clinicoradiological features in ICC with high ki67 status. (**A-D**) A 53 years old male with the history of hepatitis B, has a 22mm ICC with high ki67 status in hepatic segment IV (arrow). DWI image showed high signal intensity without target sign (**A**), axial arterial phase image showed rim enhancement (**B**), portal vein phase image (**C**) and delayed phase image (**D**) show gradual and filling enhancement.

**
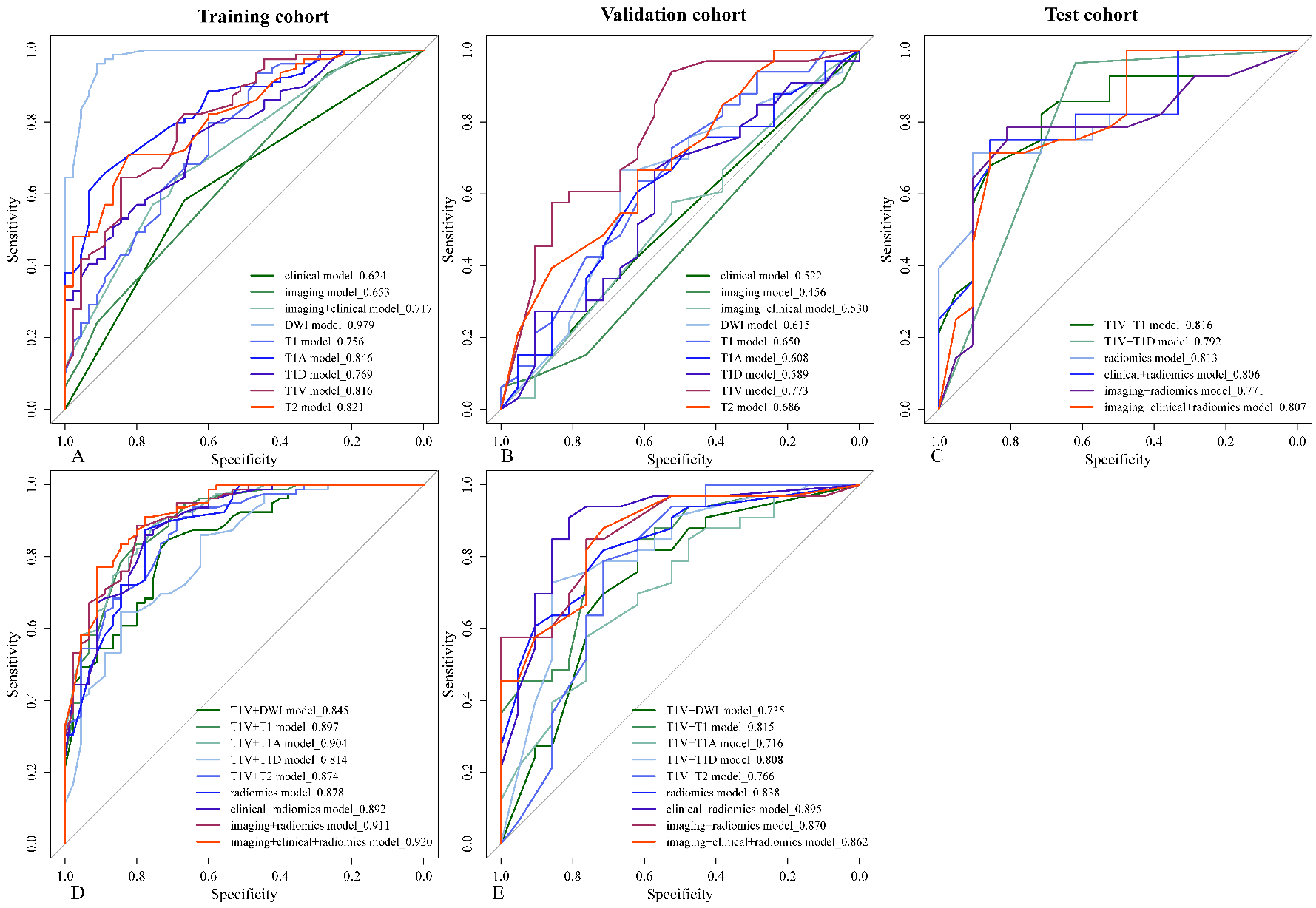
**

**Supplemental Figure 3.** Comparison of receiver operating characteristics (ROC) curves for Ki67 status prediction in training (**A, D**), validation (**B, E**) and test (**C**) cohorts by random forest.
